# GJB2 Promotes HCC Progression by Activating Glycolysis Through Cytoplasmic Translocation and Generating a Suppressive Tumor Microenvironment Based on Single Cell RNA Sequencing

**DOI:** 10.1002/advs.202402115

**Published:** 2024-08-20

**Authors:** Hanyuan Liu, Xiao Li, Chenwei Zhang, Xiaopei Hao, Yongfang Cao, Yuliang Wang, Hao Zhuang, Na Yu, Tian Huang, Chuan Liu, Hengsong Cao, Zhengqing Lu, Jinhua Song, Li Liu, Hanjin Wang, Zhouxiao Li, Weiwei Tang

**Affiliations:** ^1^ Department of General Surgery Nanjing First Hospital Nanjing Medical University Nanjing 210000 China; ^2^ Hepatobiliary Center, The First Affiliated Hospital of Nanjing Medical University Key Laboratory of Liver Transplantation, Chinese Academy of Medical Sciences NHC Key laboratory of Hepatobiliary cancers Nanjing Medical University Nanjing 210000 China; ^3^ Department of Hepatobiliopancreatic Surgery The Affiliated Cancer Hospital of Zhengzhou University & Henan Cancer Hospital Zhengzhou 450000 China; ^4^ Department of Immunology Key Laboratory of Immune Microenvironment and Diseases NHC Key Laboratory of Antibody Technique Jiangsu Key Lab of Cancer Biomarkers Prevention and Treatment Collaborative Innovation Center for Personalized Cancer Medicine Nanjing Medical University Nanjing 210000 China; ^5^ Department of Pharmaceutical Preparation General Hospital of Ningxia Medical University Yinchuan Ningxia 750004 China; ^6^ First Teaching Hospital of Tianjin University of Traditional Chinese Medicine Tianjin 301617 China; ^7^ National Clinical Research Center for Chinese Medicine Acupuncture and Moxibustion Tianjin 301619 China; ^8^ Department of plastics Shanghai Ninth People's Hospital Shanghai Jiao Tong University School of Medicine Shanghai 200011 China

**Keywords:** GJB2, glycolysis, immunotherapy, NF‐κB, PD1, scRNA‐seq

## Abstract

Despite substantial breakthroughs in the treatment of hepatocellular carcinoma (HCC) in recent years, many patients are diagnosed in the middle or late stages, denying them the option for surgical excision. Therefore, it is of great importance to find effective therapeutic targets of HCC. In this study, it is found that Gap junction protein beta‐2 (GJB2) is highly enriched in malignant cells based on single‐cell RNA sequencing and higher expression of GJB2 indicates a worse prognosis. The localization of GJB2 in HCC cancer cells is changed compared with normal liver tissue. In cancer cells, GJB2 tends to be located in the cytoplasm and nucleus, while in normal tissues, GJB2 is mainly located on the cell membrane. GJB2 is related to glycolysis, promoting NF‐κB pathway via inducing the ubiquitination degradation of IκBa, and activating HIF‐1α/GLUT‐1/PD‐L1 pathway. In addition, GJB2 knockdown reshapes tumor immune microenvironment and Salvianolic acid B inhibits the activity of GJB2. In conclusion, GJB2 promotes HCC progression by activating glycolysis through cytoplasmic translocation and generating a suppressive tumor microenvironment. Salvianolic acid B inhibits the expression of GJB2 and enhances the sensitivity of anti‐PD1 therapy, which may provide insights into the development of novel combination therapeutic strategies for HCC.

## Graphical abstract

GJB2 is highly enriched in HCC malignant cells based on scRNA‐seq and promotes HCC progression by activating glycolysis through cytoplasmic translocation, which generates a suppressive tumor microenvironment. Salvianolic acid B effectively inhibits the activity of GJB2 and enhances the sensitivity of anti‐PD1 therapy, which may provide insights into the development of novel combination therapeutic strategies for HCC.

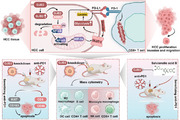

## 1 Introduction

Liver cancer, while ranking eighth in prevalence among cancer types, stands as the third leading cause of mortality.^[^ ^1^ ^]^ Notably, HCC accounts for a staggering 80% of all liver cancer instances.^[^ ^2^ ^]^ Globally, there were ≈74 7000 instances of HCC in 2019, representing a 70% increase since 1990, and the disease was responsible for 48 0000 fatalities.^[^ ^3^ ^]^ Treatment options for HCC can be quite effective, especially when diagnosed early. However, tumor recurrence remains a significant challenge. Available treatment modalities include transarterial chemoembolization (TACE), liver transplantation, ablation therapy, and surgical resection. Unfortunately, due to the asymptomatic nature of the illness, most HCC patients present in late stages. For these individuals, systemic medication is often the practical choice, as surgical alternatives may not be feasible.^[^ ^4^ ^]^

Cancer immunotherapies, particularly the use of immune checkpoint inhibitors (ICIs), have significantly transformed the clinical management of HCC in recent years Bevacizumab (anti‐VEGF) and Atezolizumab (anti‐PD1), when combined, have been demonstrated to be more effective than the first‐line therapy, sorafenib.^[^ ^5^ ^]^ Despite these advancements, HCC remains one of the most challenging cancers due to its frequent recurrence and medication resistance. Unfortunately, a substantial portion of HCC patients either experience immunological‐related side effects or do not benefit from these immunotherapies. Nevertheless, the rapid advancement of immunotherapy offers fresh hope for HCC patients, although further research is essential to enhance treatment sensitivity.

The rapidly developing single‐cell RNA sequencing (scRNA‐seq has provided profound enlightenment for genetics and tumor biology by proving its ability to characterize the single‐cell epigenome, transcriptome, and genome, empowering us to investigate the dynamic alterations that unfold across various stages of tumor growth, encompassing even the intricate landscape of advanced metastatic cancers.^[^ ^6^ ^]^ In addition, the clinical application of scRNA‐seq can profoundly change the way we treat cancer. Indeed, scRNA‐seq has ushered in a new era of biological exploration. Despite the ongoing challenges, we remain steadfast in our conviction that the capabilities showcased by this technology will persistently fuel innovation and furnish solutions to existing conundrums. In doing so, it promises to illuminate the intricate tapestry of diseases, enriching our comprehension and paving the way for transformative breakthroughs.^[^ ^7^ ^]^ What we must mention here is that based on the development of scRNA‐seq technology, the elucidation of tumor microenvironment (TME) has made great progress, and many new targets have been found. In the present study, based on scRNA‐seq data, we found that GJB2 is specific enriched in malignant cells, rather than other cells such as immune cells. It is interesting that the localization of GJB2 in HCC cancer cells was changed compared with normal liver tissue. In cancer cells, GJB2 tends to be located in the cytoplasm and nucleus, while in normal tissues, GJB2 is mainly located on the cell membrane. GJB2 is closely related to glycolysis, promoting NF‐κB pathway, activating HIF‐1α/GLUT‐1, and increasing PD‐L1 expression. Co‐immunoprecipitation (Co‐IP) results showed that GJB2 promotes the ubiquitination degradation of IκBa by recruiting ASB2 protein, and then activates NF‐κB pathway. The results of mass cytometry showed that GJB2 knockdown reshapes TME and is more beneficial to anti‐PD1 therapy. In addition, we performed a virtual screen to obtain Salvianolic acid B, a small molecule inhibitor of GJB2, and validated its ability to inhibit HCC growth in vitro and in vivo.

## 2 Results

### 2.1 GJB2 was Specifically Enriched in Tumor Cells in HCC Based on scRNA‐Seq

**Figure 1:**
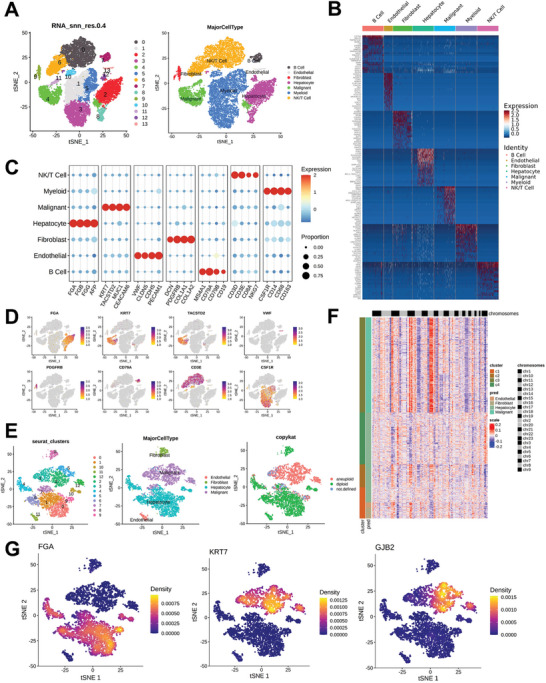
GJB2 was specifically enriched in tumor cells based on scRNA‐seq. A) According to the definition of classification of specific gene markers using tSNE plot, GSE166635 results indicated that a total of seven cell clusters in HCC (B, endothelial, fibroblast, hepatocyte, malignant, myeloid, and NK/T cells). B) The heat map shows the specific genes expressed by the cell clusters in Figure 1A. C) The dot plot shows the specific genes that define different cell clusters in Figure 1A. D) The tSNE plot shows the detailed distribution of the characteristic proteins of each cell cluster. E) All non‐immune cells were extracted and then reclustered to obtain the tSNE plot after clustering. According to the new tSNE plot, Copycat prediction showed that aneuiploidy was mainly enriched in malignant cells, while heptocyte tended to be normal diploid. F) Using CopyKAT to analyze cell types, it was found that aneuploidy was mainly enriched in malignant cells. Normal diploid cell clusters were defined as normal cells, and liver cells were significantly enriched in normal diploid cell clusters. G) According to the clustering analysis in Figure 1E, the hepatocyte‐specific marker FGA was mainly enriched in hypatocyte, the tumor‐cell‐specific marker KRT7 was mainly enriched in malignant cells, and GJB2 was mainly enriched in malignant cells.

GSE166635 results indicated that a total of seven cell clusters in HCC, comprising B, endothelial, fibroblast, hepatocyte, malignant, myeloid, and NK/T‐cell were found according to the definition of classification of specific gene markers using tSNE plot and dot plot (**Figure** **1**A–C). For instance, endothelial cell specifically expresses VWF, CLDN5, CDH5, and PECAM1 (Figure 1C). B cell expresses MS4A1, CD19, CD79A and CD79B (Figure 1C). tSNE also showed the detailed distribution of signature proteins such as FGA and KRT7 in various cell clusters (Figure 1D). In addition, we extracted all the non‐immune cells and then re‐clustered them, and found that the re‐clustering results were consistent with the cell type annotation results in Figure 1A. We used CopyKAT software to analyze CNV in all non‐immune cells in HCC tissues, including endothelial cells, fibroblasts, hepatocyte, and malignant cells. In the process of analysis, endothelial cells and fibroblasts were designated as diploid cells, and then CopyKAT was used to predict CNV in hepatocyte and malignant cells. The results showed that there were obvious CNV changes in malignant cells, and hepatocyte tended to be normal diploid (Figure 1E). Aneuploidy predicted by CopyKAT was mainly enriched in malignant cells, while hepatocyte tended to be normal diploid (Figure 1F). The characteristic marker FGA of the liver was mainly expressed in hepatocyte, the main marker KRT7 of tumor cells was mainly expressed in malignant cells, and we were excited to find that GJB2 was mainly expressed in malignant cells (Figure 1G). Moreover, we analyzed GJB2 mRNA expression in different cancers and found that GJB2 was also enriched in cancer cells of intrahepatic cholangiocarcinoma and colorectal cancer (Figures S1–S3, Supporting Information). All above indicate that GJB2 is highly enriched in HCC cancer cells, but its function is unclear.

### 2.2 GJB2 Lost Its Transmembrane Domain and was Mainly Located in The Cytoplasm in HCC

To validate the scRNA‐seq results, we used TCGA data to further predict the expression of GJB2 in HCC cancer and paracancer tissues, and the results showed GJB2 mRNA was lower expressed in cancer tissues (*n* = 371) compared with normal tissues(*n* = 50) (Figure S4A, Supporting Information). This caused our confusion, and then we retrieved the TSVDB database (http://www.tsvdb.com/plot.html) and were surprised to find that GJB2 contains two exons, close to the 5 ‘end exon1 (short) and 3′ end exon2 (long). In HCC, exon1 expression is significantly lower, indicating that in HCC, GJB2 tends to lose the transmembrane structure of exon1 and enter the cytoplasm (Figure S4B, Supporting Information). We used the TCGA database to analyze exon1 expression of GJB2 and found that its expression in HCC cancer tissues was significantly lower than that in paracancerous tissue (Figure S4B, Supporting Information). Survival analysis suggested that GJB2 with low exon1 expression had worse prognosis than GJB2 with high exon1 expression in HCC (Figure S4B, Supporting Information). Next, we focused on GJB2 expression at the protein level. GJB2 expression in 109 samples of human HCC tissues and adjacent normal tissues was measured by immunohistochemistry (Figure S4C, Supporting Information), and GJB2 was found to be overexpressed in 43.1% HCC tissues compared with that in the corresponding paracancerous tissues (Table S1, Supporting Information). According to the clinicopathological features, GJB2 overexpression was positively correlated with microvascular invasion, blood vessel invasion, and macrovascular invasion, whereas it was not significantly correlated with age, gender, tumor size, and TNM classification (Table S1, Supporting Information). According to the Kaplan–Meier survival curve, HCC patients with high GJB2 expression had reduced relapse‐free survival and overall survival (Figure S4D,E, Supporting Information). These findings indicated that upregulation of GJB2 protein indicated poor prognosis in patients with HCC.

**Figure 2:**
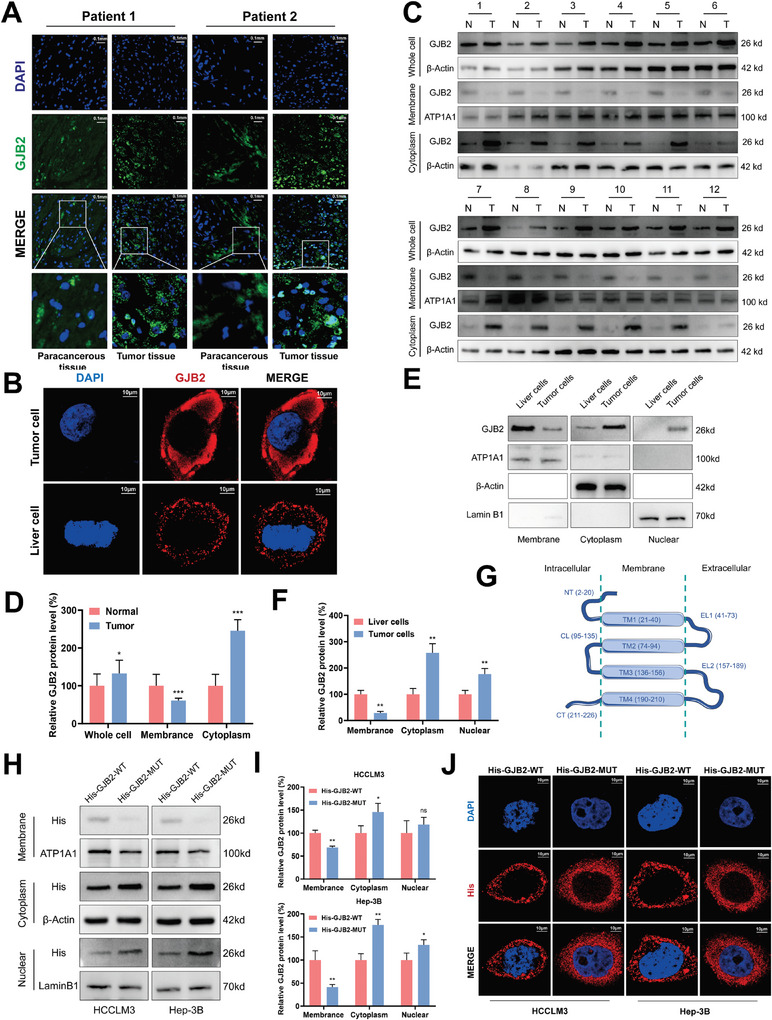
GJB2 lost its membrane segment and was mainly located in the cytoplasm in HCC. A) The representative fluorescence immunohistochemical images of puncture samples from two patients with primary HCC, Professional pathologists differentiated whether the sample was tumor tissue. (Scale bar, 0.1 mm). B) The representative immunofluorescence images of mouse HCC (Tumor cell) and liver (Liver cell) organoids. (Scale bar, 10 µm). C,D) Protein levels of GJB2 in 12 pairs of HCC tissues T) and corresponding adjacent tissues (N) were detected by Western blot analysis. *n* = 12. β‐actin is used for loading control of cytoplasmic proteins and whole‐cell proteins, and ATP1A1 is used for loading control of cytoplasmic proteins. *n* = 3 independent biological replicates. E,F) Expression of GJB2 protein in the cell membrane, cytoplasm, and nucleus of HCC cells and liver cells in mouse organoids. β‐actin is used for the loading control of cytoplasmic proteins, ATP1A1 is used for loading control of cytoplasmic proteins, and Lamin B1 is used for the loading control of nuclear protein. *n* = 3 independent biological replicates. G) Topology diagram of GJB2 protein. NT (N‐terminal domain), TM (transmembrane domain), EL (Extracellular loop), CL (Cytoplasmic loop), CT (C‐terminal domain). H,I) Expression of GJB2 protein in His‐GJB2‐WT group and His‐GJB2‐MUT group. β‐actin is used for the loading control of cytoplasmic proteins, ATP1A1 is used for the loading control of cytoplasmic proteins, and Lamin B1 is used for the loading control of nuclear protein. *n* = 3 independent biological replicates. J) The representative immunofluorescence images of His‐GJB2‐WT group and His‐GJB2‐MUT group. (Scale bar, 10 µm). In all statistical plots, data are expressed as the mean ± SD, Student's *t*‐test (Figure 2D,F,I) were used to determine statistical significance. (ns = not significant, ^*^ *p* < 0.05, ^**^ *p* < 0.01, ^***^ *p* < 0.001).

In order to further explore the changes of GJB2 transmembrane region, we used fluorescence immunohistochemical to detect the expression and localization of GJB2 protein in cancer tissue and paracancerous tissue samples from two HCC patients. We were pleased to find that GJB2 in cancer tissue was more localized in cytoplasm and nucleus, while GJB2 in paracancerous tissue was more localized in cell membrane (**Figure** **2**A), which aroused our great interest. We hypothesized that the localization of GJB2 protein was changed during carcinogenesis. To further confirm this conclusion, we also used immunofluorescence to detect the expression and localization of GJB2 protein in organoids‐cultured mouse hepatoma cells and normal liver cells. The results showed that the expression of GJB2 protein in the cytoplasm of HCC cells was higher than that of normal liver cells (Figure 2B). We used western blot to detect the expression of GJB2 in the cell membrane, cytoplasm, and overall in the paired tissues of cancer and adjacent tissues of 12 HCC patients with advanced stage, respectively. The results showed that the expression of total GJB2 protein in cancer tissues was higher than that in adjacent tissues, while the expression of GJB2 protein on the cell membrane was lower than that in adjacent tissues. The expression of cytoplasmic GJB2 protein was higher in cancer tissue than in adjacent tissues (Figure 2C,D). We also detected the expression of GJB2 protein in the cell membrane, cytoplasm, and nucleus of HCC cancer cells from mouse organoids and normal liver cells. The results showed that the expression of GJB2 protein in liver cancer cell membrane was lower than that in normal liver cells, while the expression in cytoplasm and nucleus was higher than that in liver cells (Figure 2E,F), which was consistent with the conclusion of human samples. Interestingly, we also detected the expression of CX43 from the same family of gap junction protein, but no significant differences were found (Figure S4F,G, Supporting Information).

Then, we mutated the transmembrane region of GJB2 protein, and transfected the mutant plasmid and the wild type into HCC cells respectively (Figure 2G). It was found that compared with the wild type, the expression of GJB2 protein in the mutant group was significantly reduced on the membrane, but increased in both cytoplasm and nucleus (Figure 2H,I). Immunofluorescence showed that the cytoplasm localization of GJB2 protein in the mutant group was significantly higher than that in the wild type (Figure 2J). In summary, the localization of GJB2 in HCC cancer cells has changed compared with that in normal liver tissues. In cancer cells, GJB2 tends to be located in cytoplasm and nucleus, while in normal tissues, it is mainly located in the cell membrane.

### 2.3 GJB2 Facilitated the Proliferation, Invasion and Migration of HCC In Vitro

**Figure 3:**
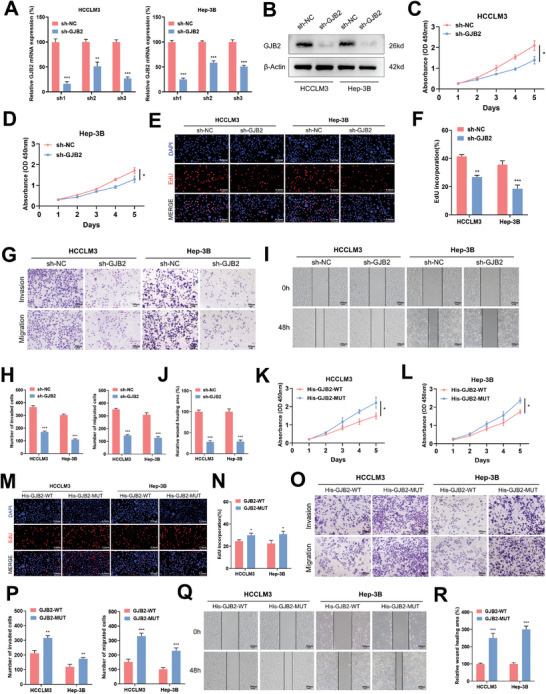
GJB2 facilitated the proliferation, invasion, and migration of human cancer cells in vitro. A) Relative mRNA expression levels of GJB2 in HCC cell lines after transfection. *n* = 3 independent biological replicates. B) Protein expression levels of GJB2 in HCC cell lines after transfection. β‐actin is used for loading control. *n* = 3 independent biological replicates. C,D) CCK‐8 assays were performed to assess the effect of GJB2 on HCC cell lines proliferation. *n* = 3 independent biological replicates. E,F) EdU assays were performed to assess the effect of GJB2 on HCC cell lines proliferation (scale bars, 0.2 mm). *n* = 3 independent biological replicates. G,H) Transwell assays were performed to determine the migration and invasion capacities of transfected HCC cell lines (scale bars, 200 µm). *n* = 3 independent biological replicates. I,J) The effects of GJB2 knockdown on HCC cell lines migration were evaluated through wound healing assays (scale bars, 200 µm). *n* = 3 independent biological replicates. K,L) The effects of His‐GJB2‐WT group and His‐GJB2‐MUT group on the proliferation of HCC cell lines were examined by cck8 assay. *n* = 3 independent biological replicates. M,N) The effects of His‐GJB2‐WT group and His‐GJB2‐MUT group on the proliferation of HCC cell lines were examined by EdU assays (scale bars, 0.2 mm). *n* = 3 independent biological replicates. O,P) Transwell assays were performed to determine the migration and invasion capacities of His‐GJB2‐WT group and His‐GJB2‐MUT group HCC cell lines (scale bars, 200 µm). *n* = 3 independent biological replicates. Q,R) The effects of His‐GJB2‐WT group and His‐GJB2‐MUT group HCC cell lines migration were evaluated through wound healing assays (scale bars, 200 µm). *n* = 3 independent biological replicates. In all statistical plots, data are expressed as the mean ± SD, Student's *t*‐test (Figure 3A,C,D,F,H,J,K,L,N,P,R) were used to determine statistical significance. (ns = not significant, ^*^ *p* < 0.05, ^**^ *p* < 0.01, ^***^ *p* < 0.001).

So, what is the function of GJB2 in cytoplasm and nucleus for HCC progression? To investigate the effect of GJB2 mRNA on HCC cells, we designed three shRNA sequences (called sh‐GJB2) that specifically target GJB2, a technique that inhibits the source of GJB2 mRNA synthesis. These sequences were employed to downregulate GJB2 expression in HCC cells. Subsequently, we assessed the expression levels of GJB2 using the qRT‐PCR method. Remarkably, our findings revealed that sh1 exhibited the highest knockdown efficiency (**Figure** **3**A). We then verified the expression of GJB2 protein with sh1 sequences, and found that the protein of GJB2 after knock‐down was lower than that of the control sh‐NC group in HCCLM3 and Hep‐3B cells (Figure 3B). In CCK8 (Figure 3C,D) and EdU (Figure 3E,F) assay, knockdown of GJB2 inhibited HCC cell proliferation. Compared with the control group in the transwell assay (Figure 3G,H) and wound healing assay (Figure 3I,J), sh‐GJB2 inhibited relative migration and invasion rate.

What is the function of GJB2 protein on HCC after loss of membrane region? We mutated the membrane protein region of GJB2 and found that compared with WT group, the cell phenotype suggested that the mutant group more promoted HCC progression, including proliferation, invasion, and migration (Figure 3K–R). These results suggest that down‐regulating the expression of GJB2, especially in cytoplasm and nucleus, can effectively inhibit the proliferation, invasion, and migration of HCC. This finding offers hope for potential therapeutic strategies targeting GJB2 in the treatment of HCC.

### 2.4 RNA Sequence and Metabolomics Analysis When GJB2 was Knocked Down in HCC Cells

**Figure 4:**
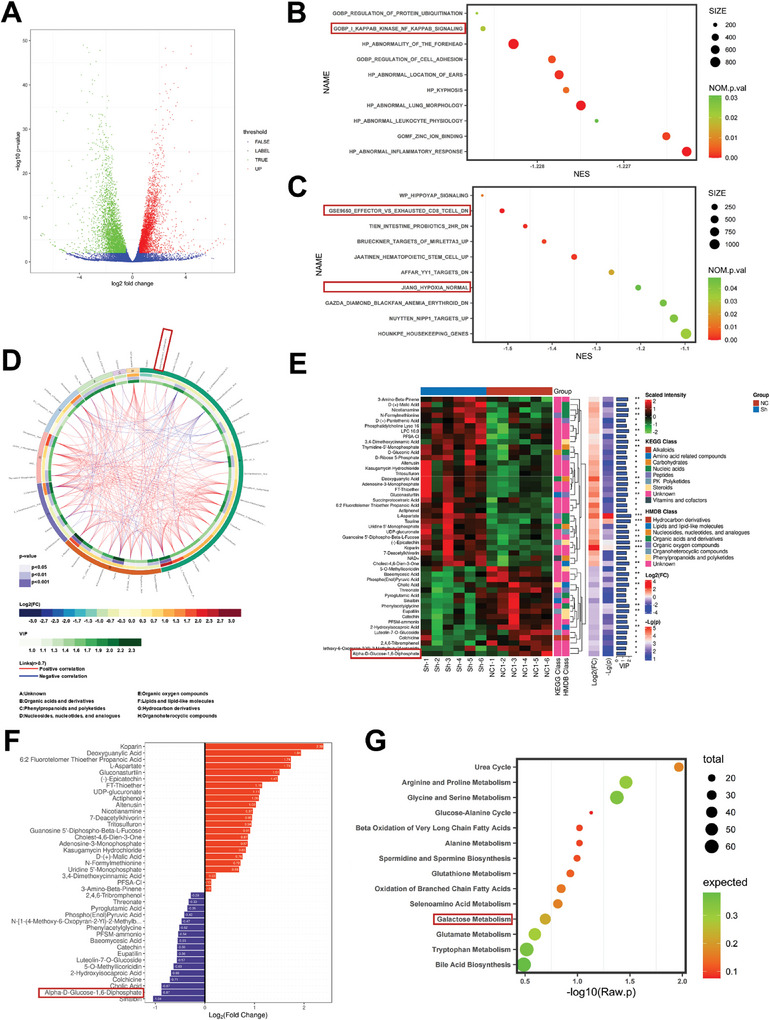
RNA sequence and metabolomics analysis of down‐regulating GJB2 in tumor cells. A) The volcano map showed differentially expressed genes after GJB2 were knockdown in HCC cells. B) According to the results of GO analysis, differentially down‐expressed genes were largely enriched in the inflammatory, protein ubiquitination, and NF‐κB pathway. C) As revealed by KEGG analysis results, CD8^+^ T exhaustion, hippo signaling, and hypoxia pathway were among the most obviously involved processes. D) The cycle diagram mainly showed the correlation between multiple differential metabolites. E) The different metabolites in each comparison group were classified and counted according to the structure and function of the metabolites, and the results of substance classification in the KEGG and HMDB databases were provided respectively. F) The top up‐regulated metabolites after GJB2 were knockdown in HCC cells. G) KEGG pathway analysis showed that these differential metabolites were mainly focused on the Glucose‐Alanine Cycle, Galactose Metabolism, Glutathione Metabolism, etc.

To reveal the complex mechanism by which GJB2 induces HCC cell progression in the cytoplasm, we embarked on an exploration using RNA sequencing. Our aim was to discern the downstream genes influenced by GJB2. The volcano map showed differentially expressed genes after GJB2 was knockdown (**Figure** **4**A). Based on the GO analysis results, it was observed that genes exhibiting differential downregulation were significantly enriched in several key pathways. Notably, these pathways included inflammation, protein ubiquitination, and the NF‐κB pathway (Figure 4B). As revealed by KEGG analysis results, CD8^+^ T exhaustion, hippo signaling, and hypoxia pathway were among the most obviously involved processes (Figure 4C). Furthermore, differentially up expressed mRNAs were analyzed as well. According to the results of GO analysis, differentially up expressed genes were enriched in the deoxyribonucleoside monophosphate catabolic process, mitochondrial dysfunction, calcium ion transport. KEGG analysis results showed TP53 mutations were among the most obviously involved processes. (Figure S5A,B, Supporting Information). In addition, gene set enrichment analysis (GSEA) suggested that the down‐regulated genes are associated with NF‐κB, CD8^+^ T exhaustion, and hypoxia pathway, consistent with the results of GO and KEGG analysis (Figure S5C–E, Supporting Information). Metabolomics enables a comprehensive and precise examination of cytokines, signaling pathways, and metabolites.^[^ ^8^ ^]^ In this study, we identified tumor cells from both the sh‐NC and sh‐GJB2 groups using metabolomics sequencing. To explore a diverse range of metabolites, we employed both mixed and negative modes. Initially, the negative mode was utilized. The central focus of the cycle diagram (Figure 4D) was the interplay between numerous differential metabolites. These metabolites were categorized and numbered based on their structure and function. The outcomes of substance categorization were obtained from the KEGG and HMDB databases (Figure 4E). The top up‐regulated metabolites (including Deoxyguanylic Acid, L‐Aspartate, Gluconasturtiin, Adenosine‐3‐Monophosphate, Adenosine‐3‐Monophosphate, etc.), and the top down‐regulated metabolites (including α‐D‐Glucose 1,6‐Bisphosphate, Threonine, Pyroglutamic Acid, Luteolin‐7‐0‐Glucoside etc.) were shown by multiplicity of differences (Figure 4F). The KEGG pathway analysis revealed that the differential metabolites primarily centered around the Glucose‐Alanine Cycle, Galactose Metabolism, Glutathione Metabolism, etc. (Figure 4G). Next, mixed patterns were analyzed and the results are shown in Figure S6 (Supporting Information). All of the metabolomic analyses indicated that the glucose metabolites are significantly altered after GJB2 is knocked down in HCC.

### 2.5 GJB2 Increased PD‐L1 by Activating Tumor Cell Glycolysis

**Figure 5:**
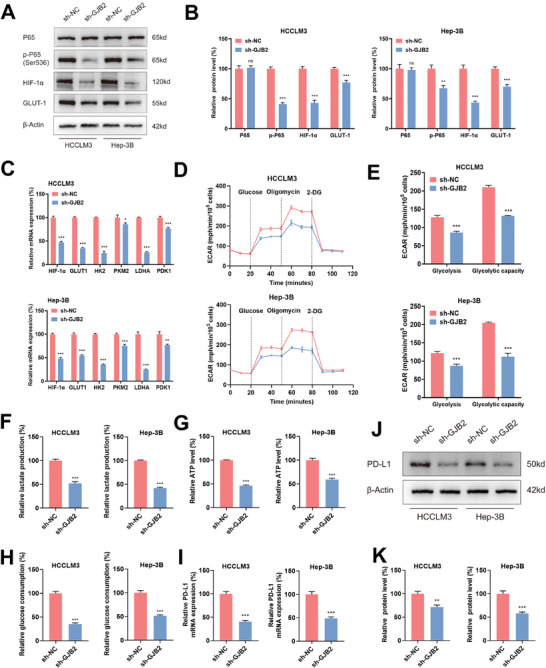
GJB2 increased PD‐L1 by activating tumor cell glycolysis. A,B) Western blot analysis to evaluate P65 p‐P65, HIF‐1α, and GLUT‐1 in lysates prepared from GJB2 knockdown HCC cells. β‐actin was used as the loading control. *n* = 3 independent biological replicates. C) qRT‐PCR analysis of HIF‐1α and key enzymes of glycolysis (GLUT‐1, HK2, PKM2, LDHA, PDK1) mRNA levels in GJB2 knockdown HCC cells relative to the sh‐NC group. β‐actin was used to normalize samples. *n* = 3 independent biological replicates. D,E) ECARs of GJB2 knockdown HCC cells were determined. According to the result of ECAR calculation Glycolysis and Glycolytic capacity. *n* = 3 independent biological replicates. F) Lactate production in GJB2 knockdown HCC cells was determined relative to the sh‐NC group. *n* = 3 independent biological replicates. G) ATP levels of GJB2 knockdown HCC cells were determined relative to the sh‐NC group. *n* = 3 independent biological replicates. H) Glucose consumption by GJB2 knockdown HCC cells was determined relative to the sh‐NC group. *n* = 3 independent biological replicates. I) qRT‐PCR analysis of PD‐L1 mRNA levels in GJB2 knockdown HCC cells relative to the sh‐NC group. β‐actin was used to normalize samples. *n* = 3 independent biological replicates. J,K) Western blot analysis to evaluate core kinases in the PD‐L1 in lysates prepared from GJB2 knockdown HCC cells. β‐actin was used as the loading control. *n* = 3 independent biological replicates. In all statistical plots, data are expressed as the mean ± SD, Student's *t*‐test (Figure 5B, C, D, E, F, G, H, I and K) were used to determine statistical significance. (ns = not significant, ^*^ *p* < 0.05, ^**^ *p* < 0.01, ^***^ *p* < 0.001).

Based on the above RNA sequencing and metabolomics results, we set out to investigate how GJB2 promotes HCC cell progression through glycolytic pathway. Previous studies have shown that the NF‐κB pathway can be used as a promoter or tumor suppressor to promote cancer progression by regulating inflammation and immune responses.^[^ ^9^ ^]^ NF‐κB can induce hypoxia inducible factor‐1α (HIF‐1α) expression and increase glucose transporter‐1 expression and glycolytic enzyme activity.^[^ ^10^ ^]^ Inspired by the literature above, we hypothesize that GJB2 activates the NF‐κB pathway, which uses HIF‐1α/GLUT‐1 to bring more glucose into HCC cells. To confirm this, we first verified the expression of P65, p‐P65, HIF‐1α and GLUT‐1 in HCC cells when GJB2 was knockdown, and the results showed that P65 did not change, while p‐P65, HIF‐1α and GLUT‐1 protein decreased significantly (**Figure** **5**A,B). qRT‐PCR showed that GLUT1, HK2, PKM2, LDHA, PDK1 and HIF‐1α were all down‐regulated when GJB2 was decreased in HCCLM3 and Hep‐3B cells (Figure 5C). ECAR revealed that the decrease in GJB2 expression inhibited the glycolysis, glycolytic ability in HCCLM3 and Hep‐3B cells (Figure 5D,E). In addition, lactic acid production, ATP levels, and glycolytic consumption were also down‐regulated when GJB2 was knocked down (Figure 5F–H).

Asgarova et al.^[^ ^11^ ^]^ have shown that PD‐L1 expression is increased by NF‐κB pathway activation and that tumor hypoxia and inflammation induced by HIF‐1α and GLUT‐1 often mediate tumor progression and cause PD‐L1 elevation. Therefore, we examined the expression of PD‐L1 after GJB2 knockdown and the results indicated that the mRNA and protein expression of PD‐L1 were significantly decreased in sh‐GJB2 group compared with the control group (Figure 5I–K).

### 2.6 GJB2 Activated NF‐κB Pathway by Promoting IκBα Ubiquitination Through Recruitment of ASB2

**Figure 6:**
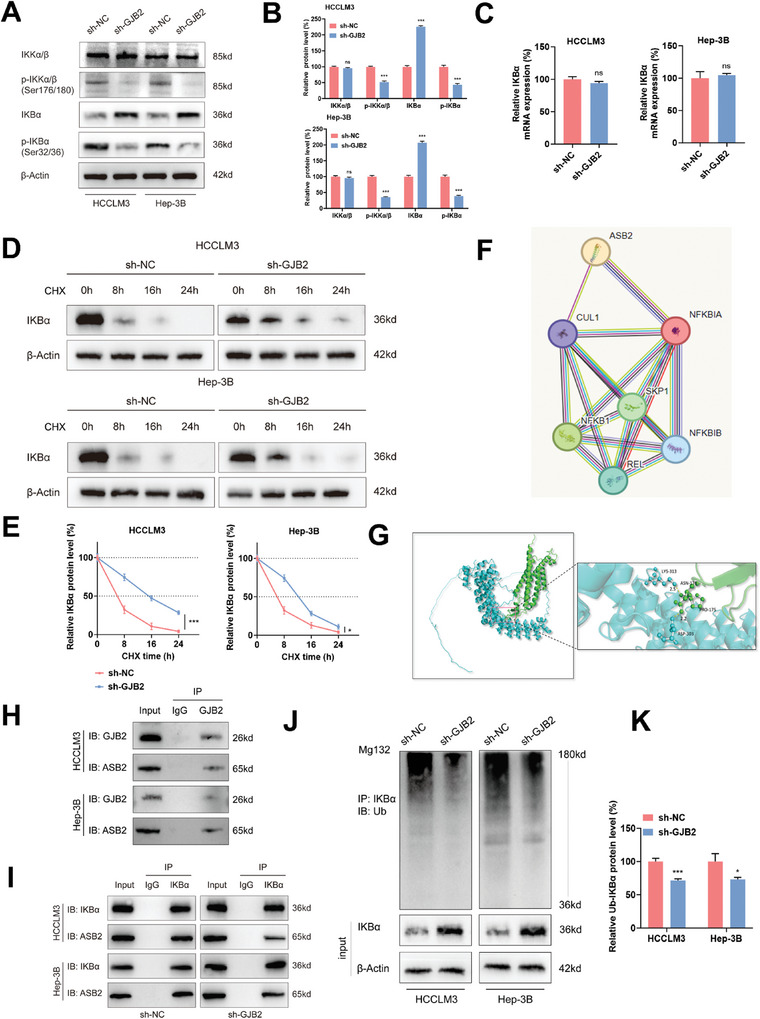
GJB2 activated NF‐κB pathway by promoting IκBα ubiquitination through the recruitment of ASB2. A,B) Western blot analysis to evaluate core kinases in the NF‐κB pathway in lysates prepared from GJB2 knockdown HCC cells. β‐actin was used as the loading control. *n* = 3 independent biological replicates. C) qRT‐PCR analysis of IκBα mRNA levels in GJB2 knockdown HCC cells relative to the sh‐NC group. β‐actin was used to normalize samples. *n* = 3 independent biological replicates. D,E) Western blot analysis of IκBα protein in GJB2 knockdown HCC cells treated with cycloheximide (CHX; 25 µg mL^−1^) for 0, 8, 16, and 24  h. Decay curve of IκBα levels normalized to β‐actin and to 0 h at the indicated time points from CHX experiments. *n* = 3 independent biological replicates. F) STRING database was used to predict that ankyrin repeats and found ASB2 protein may interact with IκBα protein. G) Artificial intelligence (AI) technology was used to docking and found that GJB2 and ASB2 proteins may also have action sites. H) Co‐IP and western blot analysis showing the interaction of GJB2 and ASB2 in HCC cell lines. I) Co‐IP and western blot analysis showing the interaction of IκBα and ASB2 in HCC cell lines after transfection. J,K) At 6  h post‐treatment with MG132 (10 µm), cell lysates were obtained and immunoprecipitated with anti‐IκBα antibody. Western blot analysis was performed with antibody to IκBα to detect endogenous IκBα ubiquitination from GJB2 knockdown HCC cells relative to the sh‐NC group. *n* = 3 independent biological replicates. In all statistical plots, data are expressed as the mean ± SD, Student's *t*‐test (Figure 6B,C,E,K) were used to determine statistical significance. (ns = not significant, ^*^ *p* < 0.05, ^***^ *p* < 0.001).

To unravel the intricate mechanisms behind the diminished NF‐κB activity in GJB2 knockdown cells, we meticulously employed protein immunoblot analysis. Our focus was on scrutinizing both the total protein levels and the phosphorylation states of core kinases intricately woven into the classical NF‐κB signaling pathway (**Figure** **6**A,B). In the GJB2 knockdown cell line, the phosphorylation levels of proteins IKKα/β (Ser176/180) and p‐IκBα (Ser32/36) were uniformly decreased. However, the protein levels of IKKα/β remained unchanged, while IκBα increased upon GJB2 knockdown. To further investigate the mechanism behind the elevated IκBα levels, we assessed IκBα mRNA levels and found no significant change following GJB2 knockdown (Figure 6C). These results suggest that IκBα protein may be stable in sh‐GJB2 group cells. Consequently, we treated GJB2‐knockdown HCCLM3 and Hep‐3B cells with Cycloheximide (CHX) to determine the half‐life of IκBα. Remarkably, the sh‐GJB2 group exhibited significantly prolonged half‐life compared to the control group (Figure 6D,E), indicating a correlation between GJB2 and IκBα stability. Based on other research findings, we hypothesize that GJB2 may activate the NF‐κB pathway by promoting ubiquitination‐mediated degradation of IκBα.

In order to further find out which ubiquitination enzyme is involved in IκBα degradation process, we used STRING database to predict that ankyrin repeats and found SOCS box containing 2(ASB2) protein may interact with IκBα protein (Figure 6F). Then, we used artificial intelligence (AI) technology to docking and found that GJB2 and ASB2 proteins may also have action sites (Figure 6G). The interaction between GJB2 and ASB2 was confirmed by Co‐IP (Figure 6H). The interaction between ASB2 and IκBα was also confirmed by Co‐IP (Figure 6I). In addition, we found that IκBα‐binding ubiquitin decreased significantly after knockdown GJB2, while IκBα expression increased (Figure 6J,K). All of these indicated that GJB2 activated NF‐κB pathway by promoting IκBα ubiquitination through the recruitment of ASB2 in HCC.

### 2.7 GJB2 Deficiency Rewired the TME and Activated Immunity in HCC

**Figure 7:**
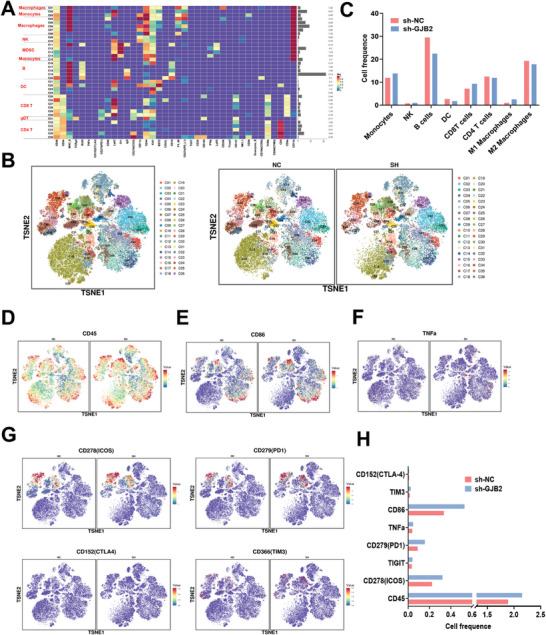
GJB2 deficiency rewired the TME and activated immunity in HCC. A) Tumor samples from mice injected with sh‐NC and sh‐GJB2 were detected subcutaneous by mass cytometry. Single, viable, and intact CD45+ immune cells were recycled from selected cells in the respective tissues. CD45+ immune cell aggregation and subgroup annotation were observed in all samples. In total, there were 36 cell clusters. B,C) The TSNE scatter plot showed the distribution of each cell cluster, and the proportion of each cell cluster was statistically analyzed. D–F) The expression of CD45, CD86, and TNF‐α was increased in the sh‐GJB2 group relative to the sh‐NC group, as defined by specific markers for the respective cell types. G) The expression of common CD8^+^T cell surface markers ICOS, PD1, CTLA4, and TIM3 increased in the sh‐GJB2 group relative to the sh‐NC group, as defined by specific markers of the respective cell types. H) Relative expression of proteins from Figure 7D–G in different groups.

To explore the changes of TME caused by GJB2 deficiency, we developed sh‐RNA targeting mouse GJB2 to knocking down mouse HCC line H22, and detected subcutaneous tumor samples from mice injected with sh‐NC and sh‐GJB2 by mass cytometry. We recycled single, viable, and intact CD45^+^ immune cells from selected cells in the respective tissues. CD45^+^ immune cell aggregation and subgroup annotation were observed in all samples. In total, 36 distinct cell clusters were identified, each defined by specific markers corresponding to the respective cell type (**Figure** **7**A; Figure S7, Supporting Information). The results showed that compared with the sh‐NC group, the relative proportion of M2 macrophages, B cells, Dendritic cells (DC), and CD4^+^ T cells in the sh‐GJB2 group decreased, while the relative proportion of monocytes, M1 macrophages, CD8^+^ T cells, and NK cells increased (Figure 7B,C). In addition, we found that CD45, CD86, and TNF‐α expression was increased in the sh‐GJB2 group (Figure 7D–F,H). However, the expression of common markers including ICOS, PD1, CTLA4, TIM3, on the surface of CD8^+^ T cells increased in the sh‐GJB2 group (Figure 7G,H), which indicated that it might be more sensitive to checkpoint inhibitors such as PD1 monoclonal antibody. These results confirm that GJB2 deficiency leads to immune activation in HCC.

### 2.8 Inhibition of GJB2 was Beneficial to Enhance the Efficacy of Anti‐PD1 Treatment in Cancer

**Figure 8:**
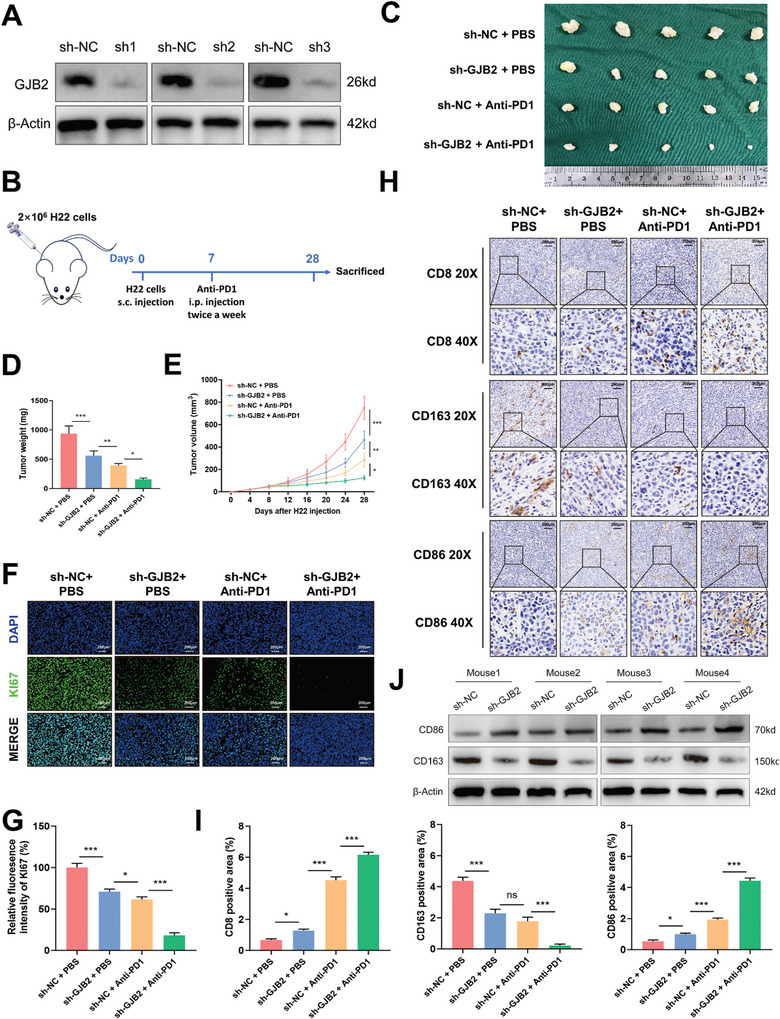
Inhibition of GJB2 was beneficial to enhance the efficacy of anti‐PD1 treatment in cancer. A) Protein expression levels of GJB2 in mouse HCC cell lines after transfection. β‐actin is used for loading control. B) Schematic of the animal model. C) Photographs of tumors induced by the subcutaneous inoculation of mice (*n* = 5 mice per group) with transfected H22 cells. D) Graphs of tumor weights. *n* = 5 mice per group. E) Growth curves of tumor volumes. *n* = 5 mice per group. F,G) Representative Ki‐67 fluorescence immunohistochemical of tumors (scale bars, 200 µm). *n* = 3 mice per group. H,I) Representative CD8, CD86, CD163 Immunohistochemistry of tumors (scale bars, 200 µm). *n* = 3 mice per group. J) Western blot analysis of the expression levels of CD86 and CD163 in tumor tissues of mice in sh‐NC group and sh‐GJB2 group. β‐actin was used as the loading control. *n* = 4 mice per group. In all statistical plots, data are expressed as the mean ± SD, one‐way ANOVA (Figure 8D,E,G,I) were used to determine statistical significance. (ns = not significant, ^*^ *p* < 0.05, ^**^ *p* < 0.01, ^***^ *p* < 0.001).

Based on the previous conclusion, it has been proved that knockdown of GJB2 can not only reduce the expression of PD‐L1, but also activate TME. We would like to try whether inhibition of GJB2 combined with anti‐PD1 can enhance the anti‐tumor effect in vivo. To confirm this idea, we used sh‐RNA to knock down GJB2 in H22 cells (**Figure** **8**A), and subcutaneous injected H22 cells from each group into C57BL/6 mice, respectively. Anti‐PD1 intraperitoneal injection was administered on day 7 and twice a week until day 28 when mice were sacrificed (Figure 8B). The results showed that the tumor volume and weight of sh‐GJB2 group were reduced compared with sh‐NC group. Moreover, sh‐GJB2+Anti‐PD1 group was significantly reduced compared with sh‐NC+Anti‐PD1 group (Figure 8C–E). Fluorescence immunohistochemical results showed that KI67 was significantly down‐regulated in the sh‐GJB2+Anti‐PD1 group compared with sh‐NC+Anti‐PD1 group (Figure 8F,G). Immunohistochemical results revealed that knockdown of GJB2 led to increased expression of CD8 and CD86, and decreased expression of CD163, which was consistent with the results of mass cytometry (Figure 8H,I). Protein expression in mouse subcutaneous tumor tissue also suggested that CD86 protein expression was increased and CD163 expression was decreased after GJB2 knockdown (Figure 8J). These results suggest that GJB2 can enhance the sensitivity of anti‐PD1 immunotherapy in the mouse subcutaneous HCC model.

### 2.9 Salvianolic Acid B, a Small Molecule Inhibitor Targeting GJB2 Could Slow HCC Progression Based on Structural Virtual Screening

**Figure 9:**
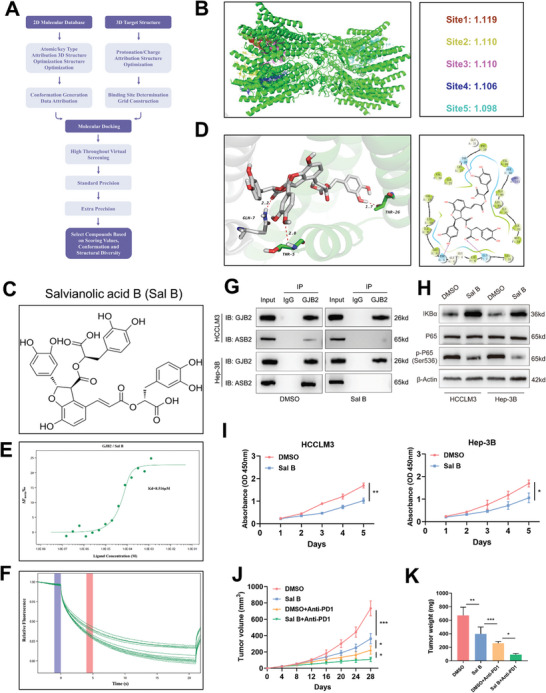
Salvianolic acid B, a small molecule inhibitor targeting GJB2 could slow HCC progression based on structural virtual screening. A) Flowchart of virtual screening. B) The active site of human GJB2 (PDB ID:7QEQ) was predicted and scored based on its 3D structure. Virtual screening was performed according to the active site Site1(key amino acid: ILE9/VAL37/LEU89) with the highest score. C) Chemical structural formula for Salvianolic acid B. D) 2D and 3D patterns of Salvianolic acid B and Human GJB2 binding. The two hydroxyl groups of Salvianolic acid B each form three hydrogen bonds with Human GJB2 protein. E,F) MST assay of the GJB2 and Salvianolic acid B. The fitted binding curve gives a Kd of 8.516 µM. G) Co‐IP and western blot analysis demonstrated the interaction of GJB2 and ASB2 after treatment of HCC cell lines with DMSO or SalB. β‐actin was used as a loading control. H) After treatment of HCC cell lines with DMSO or Salvianolic acid B (100µM), the core kinase of NF‐κB pathway was detected by Western blot. β‐actin was used as a loading control. *n* = 3 independent biological replicates. I) CCK‐8 assays were carried out to assess the effect of Salvianolic acid B (100µm) on HCC cell proliferation. *n* = 3 independent biological replicates. J) Growth curves of tumor volumes. *n* = 5 mice per group. K) Graphs of tumor weights. *n *= 5 mice per group. In all statistical plots, data are expressed as the mean ± SD, one‐way ANOVA (Figure 9J,K) and Student's *t*‐test (Figure 8I) were used to determine statistical significance. (ns=not significant, ^*^ *p* < 0.05, ^**^ *p* < 0.01, ^***^ *p* < 0.001).

In recent years, literature searches have shown partial research on the function of GJB2, but small molecule drugs designed with GJB2 as a drug target have not yet been reported. We conducted virtual screening of the 3D structure of human GJB2 (PDB ID:7QEQ) through a literature search (**Figure** **9**A). Since the active site of GJB2 is unknown, we predicted its active site and scored it. As can be seen from the Figure 9B, Site1 has the highest score and is more likely to be the active site of Human GJB2 protein. In this study, the extracellular active site Site1 (key amino acids: ILE9/VAL37/LEU89) of human GJB2 protein structure was virtually screened by computer in order to obtain small molecular compounds with strong binding ability to target proteins. After docking compounds in MCE Library with target protein Human GJB2, five optimal compounds including Militarine, Salvianolic acid B, Parishin B, VER‐155008, RGX‐104 were initially selected after comprehensive evaluation of the docking score, fat solubility, and water solubility.

The drug Salvianolic acid B (Sal B) was selected for further study (Figure 9C). 2D and 3D patterns of drug bonding showed that the two hydroxyl groups of Sal B formed three hydrogen bonds with Human GJB2 protein, respectively (Figure 9D). The two phenol hydroxyl groups could form two hydrogen bonds with THR5 and THR26 of Å chain at distances of 2.8 and 1.7 Å, respectively. The carboxyl group acts as A hydrogen bond receptor to form a hydrogen bond with GLN7 of the F chain at a distance of 2.2 Å. To demonstrate in vitro the combination of Sal B and GJB2, we used MST to measure the binding affinity Kd of Sal B to GJB2 protein. The results showed that Sal B was able to assemble with GJB2, with a dissociation constant (Kd) of 8.516 µM for binding affinity (Figure 9E,F). The Co‐IP results showed that the protein binding rate of GJB2 and ASB2 was weakened after Sal B was added (Figure 9G). In western blot assay, Sal B was found to inhibit NF‐κB pathway in tumor cells (Figure 9H). In CCK8 (Figure 9I) and EdU (Figure S8A,B, Supporting Information) assay, Sal B inhibited HCC cell proliferation. Compared with the control group in the transwell assay (Figure S8C,D, Supporting Information) and wound healing assay (Figure S8E,F, Supporting Information), Sal B inhibited relative migration and invasion rate. These results suggested that Sal B could inhibit the proliferation, invasion, and migration of HCC. We injected H22 cells into C57BL/6 mice. Sal B injection and anti‐PD1 were then performed on day 7 to assess its anti‐tumor ability. The results showed a significant decrease in tumor volume and weight in the Sal B+anti‐PD1 group compared with the anti‐PD1 group (Figure 9J,K). The above drug validation results indicate that Sal B can enhance the sensitivity of anti‐PD1 immunotherapy by inhibiting activity ofGJB2, which confirms its promoting effect on cancer immunotherapy and potential for clinical transformation in HCC.

## 3 Discussion

GJB2, commonly referred to as Connexin 26 (Cx26), belongs to the gap junction protein family. These proteins play a crucial role in the development of gap junction channels and hemichannels. When hemichannels open, signaling molecules such as glutamate and ATP can be released into the extracellular environment. Gap junctional intercellular communication (GJIC) facilitates the exchange of ions and physiologically active molecules, including second messengers, between adjacent cells that are in direct contact.^[^ ^12^ ^]^ GJIC plays a crucial role in cellular differentiation, proliferation, and apoptosis. Dysregulation of GJIC is directly associated with oncogenesis.^[^ ^13^ ^]^ In various malignancies affecting the breast, colon, lung, and cervical regions, abnormal expression of the GJB2 gene disrupts GJIC.^[^ ^14^, ^15^ ^]^ A comprehensive pan‐cancer study conducted by Yuting Jia and colleagues^[^ ^16^ ^]^ explored the potential significance of GJB2 in prognostic prediction and cancer immunotherapy response. Their findings revealed that a wide spectrum of cancers exhibited elevated expression levels of the GJB2 gene. Furthermore, GJB2 expression correlated either positively or negatively with survival outcomes in several malignancies. Remarkably, the expression levels of GJB2 correlate with tumor mutational burden, microsatellite instability, neoantigens, and immune cell infiltration within the TME. These findings underscore the pivotal role of GJB2 in shaping the TME. Functional enrichment analysis revealed that GJB2, a gene implicated in tumor biology, plays a multifaceted role in various cancer types. The multifaceted functions of this entity encompass several critical roles. It orchestrates the modulation of gap junction‐mediated intercellular transport, fine‐tunes cell communication via electrical coupling, facilitates ion transmembrane transport, actively participates in autocrine signaling pathways, exerts influence on apoptotic signaling cascades, contributes to NOD‐like receptor signaling, and significantly impacts both the p53 and PI3K‐Akt signaling pathways. In our ongoing investigation, we have discerned a notable upregulation of GJB2 within malignant cells. This heightened expression is closely associated with the promotion of HCC proliferation, invasion, and metastasis, as substantiated by scRNA‐seq data. A higher expression of GJB2 indicated a worse prognosis. These compelling findings underscore GJB2's candidacy as a promising therapeutic target in the realm of cancer research.

The biggest innovation of this study is that the localization of GJB2 in HCC cancer cells is changed compared to normal liver tissue. In cancer cells, GJB2 tends to be located in the cytoplasm and nucleus, while in normal liver cells, GJB2 is mainly located on the cell membrane. We mutated the membrane protein region of GJB2 and found that compared with the WT group, the cell phenotype suggested that the mutant group more promoted HCC progression, including proliferation, invasion, and migration. Praveena S Thiagarajan et al. found that GJB2 is elevated in self‐renewing cancer stem cells (CSCs) and is necessary and sufficient for their maintenance in triple‐negative breast cancer (TNBC). In TNBC, GJB2 is not expressed in the plasma membrane where connexins form GJs or connexons, but was enriched in the cytoplasmic fraction.^[^ ^17^ ^]^ This result is highly consistent with the conclusion of our study, reflecting the subcellular localization changes of GJB2 in the process of cancer promotion in order to adapt to tumor survival. However, we did not do further research on the root cause of the subcellular localization of GJB2 protein, and we hope to have further reports in the future.

Warburg effect or aerobic glycolysis is a metabolic mode unique to tumor cells, which preferentially metabolize to lactate by glycolysis even in the presence of sufficient oxygen and good mitochondrial function.^[^ ^18^, ^19^ ^]^ From a conventional perspective, the excess lactate associated with the Warburg effect might seem like an inefficient use of cellular resources, but it has been shown that the ATP produced by glycolysis is sufficient for tumor cells to grow. If tumor cells use most of their glucose to produce ATP through oxidative phosphorylation, that would not be sufficient to synthesize Acetyl‐coA and NADPH, which are essential for cell proliferation, explaining the important metabolic demand for tumor cells to choose aerobic glycolysis. In this study, we found that GJB2 activates the NF‐κB pathway, which uses HIF‐1α/GLUT‐1 to bring more glucose into HCC cells. Results showed that P65 did not change, while p‐P65, HIF‐1α, and GLUT‐1 protein decreased significantly. qRT‐PCR showed that GLUT1, HK2, PKM2, LDHA, PDK1 and HIF‐1α were all down‐regulated when GJB2 was decreased in HCCLM3 and Hep‐3B cells. In addition, ATP levels, glycolytic consumption, and lactic acid were also down‐regulated when GJB2 was knocked down. Many studies have linked NF‐κB to glycolysis. Jordi Rius et al.^[^ ^20^ ^]^ demonstrated that NF‐κB is a key transcriptional activator of HIF‐1α, and with the phosphorylation of NF‐κB, HIF‐1α encodes VEGF and GLUT‐1 in a hypoxia‐dependent manner. GLUT‐1 can transport glucose into the cell, which in this way can provide sufficient energy to the tumor cells and undergo metabolic reprogramming, and the conversion of both glucose and glutamine to lactate involves LDH. In addition, we demonstrated the detailed process of activation of NF‐κB pathway by GJB2. The interaction between ASB2 and IκBα was confirmed by immunoprecipitation. In addition, we found that IκBα‐binding ubiquitin decreased significantly after knockdown, while IκBα expression increased. There was no significant difference in p‐IKKα binding ubiquitin. Collectively, these observations converge on the activation of the NF‐κB pathway by GJB2. This activation mechanism involves the facilitation of IκBα ubiquitination, orchestrated through the recruitment of ASB2 within HCC. Notably, ASB2, a constituent of the ankyrin repeat and SOCS box‐containing (ASB) protein family, operates as a pivotal subunit within a multifaceted E3 ubiquitin ligase complex. Giulio Sartori et al.^[^ ^21^ ^]^ reported that FLI1 regulates both the classical NF‐κB pathway at the transcriptional level, and the alternative NF‐κB pathway, via ASB2 in Diffuse large B‐cell lymphoma (DLBCL). The diminution of the ASB2 gene proved deleterious in DLBCL cell lines, precipitating the inhibition of NF‐κB through the concurrent downregulation of RelB and the augmentation of IκBα. Intriguingly, the suppression of FLI1, but not ASB2, instigated a decrease in NF‐κB1 and RelA protein levels. For the first time, Wu Wei et al.^[^ ^22^ ^]^ demonstrated that Notch1 may trigger ASB2 transcription, which subsequently activates NF‐κB in T cell acute lymphoblastic leukemia (T‐ALL) cells. Asb2α leads to the degradation of IκBα, causing it to dissociate from NF‐κB within T‐ALL cells. Moreover, targeted inhibition of Asb2α expression can induce cell death and prevent T‐ALL cell proliferation. Our study is corroborated by existing reports. However, our work represents the first global report revealing that GJB2 promotes IκBα degradation through ASB2 recruitment.

**Figure 10:**
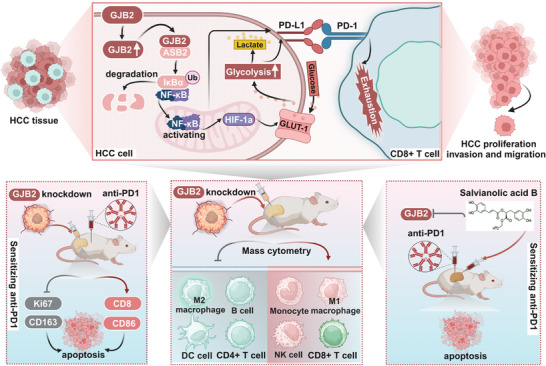
Pattern diagram GJB2 promotes the ubiquitination degradation of IκBa by recruiting ASB2 protein, and then activates NF‐κB pathway, which brings more glucose into cancer cells by activating HIF‐1α/ GLUT‐1, and increases the transcription of PD‐L1. GJB2 knockdown reshapes TME and is more beneficial to anti‐PD1 therapy in HCC (Created with BioRender.com).

Another highlight of our research is the discovery that GJB2 promotes the rise of PD‐L1 by activating the glycolytic pathway leading to immune escape in HCC. T lymphocytes play a crucial role as the primary effector cells in the anti‐tumor immune response.^[^ ^23^ ^]^ However, within the TME, there exists an abundance of immune‐suppressive cells and molecules. These factors can induce the upregulation of immune checkpoint receptors on T cells, leading to the loss of their anti‐tumor functionality and triggering immune escape by tumor cells. Commonly encountered immune‐suppressive molecules include CTLA‐4, PD‐(L)1, LAG‐3, and TIM‐3. PD‐(L)1 has emerged as a research hotspot in recent years.^[^ ^24^ ^]^ In HCC, PD‐L1 is predominantly manifested in neoplastic cells and specific immune‐suppressive cells, notably myeloid‐derived suppressor cells (MDSCs) and tumor‐associated macrophages (TAMs). The mechanisms underlying PD‐L1 upregulation are multifaceted.^[^ ^25^ ^]^ Early studies implicated various transcription factors, including HIF‐1α and IRFs, in the regulation of PD‐L1. More recent research has highlighted the role of epigenetic modifications and post‐translational regulation in controlling PD‐L1 expression.^[^ ^26^ ^]^ In our own investigation, we discovered that GJB2 activates NF‐κB, leading to increased PD‐L1 expression. Furthermore, elevated levels of HIF‐1α and GLUT‐1, induced by tumor hypoxia and inflammation, often mediate tumor progression and further enhance PD‐L1 expression. While similar studies exist, our approach involved a detailed analysis of the overall impact of GJB2 knockdown on the TME using mass cytometry rather than focusing solely on specific cell types (**Figure** **10**A).

Mass cytometry facilitates meticulous immunophenotyping of cellular populations, exhaustive examination of intracellular signaling networks, scrutiny of functional interconnectivity among cell subsets, and high‐throughput, multiparameter detection of an extensive array of samples.^[^ ^27^ ^]^ We found that the proportion of monocytes, M1 macrophages, CD8^+^ T cells, and NK cells was increased when GJB2 was knockdown. These results confirm that GJB2 deficiency leads to immune activation in HCC. Literature reports suggest that an abundance of CD8^+^ T cells and M1 macrophages, along with a reduction in M2 macrophages within the TME, favor increased sensitivity to immunotherapy.^[^ ^28^, ^29^ ^]^ To further confirm this, we used sh‐RNA to knock down GJB2 in H22 cells, and injected H22 cells from each group into C57BL/6 mice, respectively. The findings revealed a significant reduction in both the tumor volume and weight of the sh‐GJB2+anti‐PD1 group when juxtaposed with the sh‐NC+anti‐PD1 group. These observations imply that GJB2 may potentiate the sensitivity of anti‐PD1 immunotherapy in HCC. This conclusion brings good news for HCC patients who are not sensitive to anti‐PD1 treatment. Inhibition of GJB2 can not only kill cancer cells, but also reshape the microenvironment, which is conducive to the treatment of PD1 monoclonal antibody in HCC.

Predicated on the anti‐neoplastic efficacy of GJB2, as indicated by the preceding experiments, the drugs that inhibit GJB2 activity could pave the way for a novel stratagem in cancer immunotherapy. Computer‐assisted drug discovery (CADD) studies the properties of molecules to develop new therapeutic solutions through computational tools and data resources. In its broadest sense, it includes computational methods for designing or selecting compounds as potential candidates before synthesis and testing for their biological activity.^[^ ^30^ ^]^ We performed a virtual screening of the 3D structure of Human GJB2 (PDB ID:7QEQ), obtained 200 compounds in the MCE library, and selected Salvianolic acid B based on a comprehensive assessment of active site score, docking score, lipid solubility, and water solubility. Natural products and traditional Chinese medicine (TCM) are currently receiving attention as potential cancer treatments. Sal B was isolated from Danshen, an herbal medicine, and is being used as a quality control ingredient and active marker for Danshen products. Sal B, endowed with 18 phenolic hydroxyl groups, has been discovered to have a close association with redox potential and antioxidant activity. Contemporary research in oncology indicates that Sal B harbors the potential to impede the proliferation of neoplastic cells across a diverse array of malignancies, including but not limited to, head and neck squamous cell carcinoma, non‐small cell lung cancer, and breast cancer.^[^ ^31^, ^32^ ^]^ In our study, we found Sal B may be a new potential drug targeting GJB2 and provides new ideas for enhancing the effect of immunotherapy in HCC.

## 4 Conclusion

GJB2 is highly enriched in HCC malignant cells based on scRNA‐seq and higher expression of GJB2 protein indicated a worse prognosis. Mechanically, GJB2 promotes HCC progression by activating glycolysis pathway through cytoplasmic translocation and generating a suppressive TME. Salvianolic acid B effectively inhibits the activity of GJB2 and enhances the sensitivity of anti‐PD1 therapy, which may provide insights into the development of novel combination therapeutic strategies for HCC (Figure 10).

## 5 Experimental Section

### Single‐Cell RNA Data Analysis

This data was sourced from the GEO database GSE166635 and investigated the differential expression of GJB2 mRNA in HCC tissues. Single cells were selected for further study based on the following criteria: UMI counts between 3000 and 40 000; and mitochondrial percentages less than 10% of the overall UMI count. It was transformed from the standardized gene expression scale (in UMI) to LOG2 (UMI + 1). A total of 25 189 single‐cell transcriptomes from tumor tissues of two HCC patients were obtained after initial quality controls. According to the definition of cell clusters based on the specific genetic markers of different cells, GSE166635 results showed that HCC had a total of seven cell clusters (B cells, endothelial cells, fibroblasts, hepatocytes, malignant cells, myelocytes, and NK/T cells). The cell clusters were re‐analyzed with CopyKAT to distinguish malignant cells from other cells according to chromosome copy number variation (CNV). In addition, GJB2 mRNA expression from GSE138709, EMTAB8107, and GSE166555 datasets was analyzed.

### Cells and Cell Culture

Mouse HCC cells (H22) and human HCC cells (Hep‐3B and HCCLM3) were supplied by the Cell Bank of Type Culture Collection. H22 cells were cultured with RPMI 1640 medium (Gibco, USA), Hep‐3B and HCCLM3 cells were cultured with DMEM medium (Gibco, USA). Under controlled conditions of 37 °C and 5% CO₂ within an incubator, all cells were cultured in a medium containing 10% fetal bovine serum (Gibco, USA) and 1% penicillin/streptomycin (Gibco, USA).

### Patients and Tissue Specimen Collection

Adhering to the principles of the Helsinki Declaration, patients were duly informed about the details of this research. Following their informed consent, HCC tissues were procured from patients undergoing surgical therapy or preoperative puncture at the First Affiliated Hospital of Nanjing Medical University and the Affiliated Cancer Hospital of Zhengzhou University & Henan Cancer Hospital. This study, which involves human participants, received approval from the Ethics Committee of Nanjing Medical University and Zhengzhou University (2019‐SRFA‐238, 2020‐SRFA‐314). The tumor specimens in question were meticulously verified and categorized by seasoned clinicians.

### RNA Extraction and qRT‐PCR

The Cell/Tissue Total RNA Isolation Kit (Vazyme, China) to extract total RNA from both tissues and cells was employed. Subsequently, the RNA into cDNA using a reverse transcription kit (also from Vazyme, China) was converted. A comprehensive list of all primer sequences can be found in Table S2 (Supporting Information). To ensure accurate quantification, the levels of mRNA expression using the internal control, β‐Actin were normalized.

### Cell Transfection

GJB2 expression was downregulated in mouse and human HCC cell lines by using shRNA (Genechem, China). For a whole day, 1 × 10^5^ cells in each well of a six‐well plate with 2 mL of medium were incubated. Subsequently, the medium was increased to 1 mL, and 40 µL of polybrene (Sigma‐Aldrich, USA) was combined with the relevant quantity of virus. Following a 12‐ to 16 h incubation period, the cells were cultivated in standard media, and puromycin was added for screening (Beyotime, China). Table S2 (Supporting Information) displays the sequences of the shRNA that were created using the ingredients. qRT‐PCR and Western blotting assays to monitor the transfection effectiveness were used. ≈24 h before transfecting the plasmid, HCCLM3, and Hep‐3B were passaged with a seeding density of about 1 × 10^5^ cells/well. Transfection was performed when the cell confluence reaches 70%. According to the manufacturer's instructions, a mixture of Lipomaster 3000 (Vazyme, China), mutant plasmid, and opti‐MEM medium was prepared and left to stand for 10 min before being added to the culture dish in proportion. After 12 h post‐transfection, the culture medium was replaced with fresh medium, and cells were collected 48 h after transfection for subsequent experiments. The sequence of the mutant plasmid can be found in Table S2 (Supporting Information).

### Cell Proliferation Assay

Both HCC cells were independently plated in 96‐well plates and categorized into test and control groups. To plate 1000 cells, 100 µL of media in each well and incorporated 10 µL of CCK‐8 solution (RiboBio, China) was utilized. A microplate reader was used to measure cell absorbance (OD) at 450 nm at 1, 2,3, 4, and 5 days of culture following the guidelines provided by the manufacturer (Synergy, USA). For evaluation of cellular proliferation capacity, Cell‐Light 5‐ethynyl‐2′‐deoxyuridine (EdU) assays with the EdU DNA Cell Proliferation Kit (RiboBio, China) were performed. In a 24‐well plate, each well was populated with 50 000 cells. Post‐regular culture, cells were exposed to 50 mmol L^−1^ EdU solution for a period of 2 h and subsequently fixed with 4% paraformaldehyde. In accordance with the kit's instructions, the cellular strains with Apollo Dye Solution and DAPI, followed by imaging and enumeration using an Olympus FSX100 microscope (Olympus, Japan) were treated.

### Transwell Assay

By adhering to the manufacturer's guidelines, HCC cells were placed in the upper chambers with 200 µL of serum‐free RPMI 1640 medium (Gibco, USA). In preparation for the invasion and migration assays, the transwell chamber (Corning, USA) was coated with a matrigel mixture (BD Biosciences, USA). The bottom chamber was filled with RPMI 1640 medium (Gibco, USA) and 10% FBS (Gibco, USA) to serve as a cell attractant. Following a 48 h incubation period, the top chambers were fixed and stained with crystal violet dye (Beyotime, China) for 15 min. Cell counting and imaging were conducted in three different fields for visualization purposes.

### Wound Healing Assay

HCC cells were evenly distributed in six‐well culture plates. A standard 20 µL pipette tip was employed to create deliberate wounds on the confluent cell layer. The controlled elimination process aimed to segregate debris and floating cells at the well's base. Subsequently, a serum‐free medium was introduced, and the plate was subjected to a 37 °C incubation. The width of the scratch was measured using an inverted microscope and images were captured at 0 and 48 h. This experimental procedure was repeated thrice to assess both the initial wound width and the extent of cell migration.

### Immunofluorescence and Immunohistochemistry

Time of 24 h in advance, HCCLM3, and Hep‐3B are passaged, and the cells are evenly inoculated on sterile cover glasses placed in a six‐well plate. After overnight incubation, the cell confluence is allowed to reach 70%. After washing the cells with PBS, they are fixed with 4% paraformaldehyde for 10 min. After washing the cells with PBS again, a blocking solution (Beyotime, China) is added and incubated for 60 min. The primary antibody (His, GJB2) is then added after dilution and incubated overnight at 4 °C. After 24 h, the cells are washed three times with TBST, and then fluorescent secondary antibodies (Beyotime, China) are added and incubated for 1 h. After the incubation is complete, typical images are captured using a confocal microscope (Leica, Germany).

In the process of conducting immunohistochemistry, the initial step involved fixing the sample at room temperature using a 4% solution of paraformaldehyde for a duration of 20 min. Subsequently, the sample was subjected to immersion in a 0.05% solution of Triton X‐100 for a period of 5 min. Following these treatments, the samples were sealed overnight in a solution containing 2% BSA PBS and were exposed to primary antibodies (GJB2) at a temperature of 4 °C. The treated samples were then linked with either Alexa Fluorite or HRP (Beyotime, China) at room temperature. A combined secondary antibody was applied and left to incubate for 1 h. To maintain the integrity of the nucleus, DAPI staining (Beyotime, China) was utilized. Subsequent to drying, images were captured utilizing a confocal microscope (Leica, Germany). A detailed list of the antibodies employed in this investigation can be found in Table S3 (Supporting Information).

### Western Blotting

Whole cell proteins were extracted from the cells using RIPA lysis buffer (NCM, China) supplemented with protease and phosphatase inhibitors (Beyotime, China). Membrane proteins, nuclear proteins, and cytoplasmic proteins were extracted using the membrane protein, nuclear protein, and cytoplasmic protein extraction kit (KeyGen, China) and according to the manufacturer's instructions. After protein extraction, the appropriate loading buffer (Beyotime, China) was added, heated in 99 °C water bath for 5 min, and stored at −80 °C. The proteins were separated by 10% SDS‐PAGE (NCM, China), and transferred to PVDF membranes (Millipore, USA) as per the manufacturer's operating manual. After blocking the membranes for 30 min with blocking buffer (NCM, China), the membranes were incubated with primary antibodies for a whole night at 4 °C. The ECL signals were seen using an ECL Kit (NCM, China) following a 1 h incubation period with the corresponding secondary antibodies and three TBST‐buffered saline washings every 10 min. Antibodies used in this study are listed in Table S3 (Supporting Information). Different loading control is used according to different protein samples. Whole‐cell proteins and cytoplasmic proteins use β‐actin as a loading control, membrane proteins use ATP1A1 as a loading control, and nuclear proteins use Lamin B1 as a loading control.A detailed list of the antibodies employed in this investigation can be found in Table S3 (Supporting Information).

### Metabolomics Sequencing and Analysis

Ultrasound treatment with 1 mL of pre‐cooled acetonitrile/methanol/water (v/v, 2:2:1) in an ice bath for 1 h to extract metabolites from cell residues. Incubate the mixture at −20 °C for 1 h, then centrifuge at 14 000 g, 4 °C for 20 min, and transfer to a sampling vial for LC‐MS analysis. Using the UPLC‐ESI‐Q‐Orbitrap‐MS system (UHPLC, Shimadzu Exera X2 LC‐30AD Shimadzu, Japan) and Q‐Exactive Plus (Thermo Scientific, USA) coupling analysis of metabolomics profiles. Quality control (QC) samples were meticulously prepared by combining equal portions of all samples that represented the analytical sample. These QC samples were served as a crucial reference for data normalization. Throughout the collection process, blank samples (consisting of 75% ACN in water) and QC samples were introduced, injecting them at regular intervals of every six samples. Use MS‐DIAL to process raw MS data for peak comparison, retention time correction, and peak area extraction. Metabolites were identified through precise quality (quality tolerance <0.01 Da) and MS/MS data (quality tolerance <0.02 Da), which were matched with public databases such as HMDB and Massbank, as well as own metabolic standard library. KEGG pathway analysis of differential metabolite data using KEGG database (http://www.kegg.jp). Fisher's precise experiment was used for KEGG enrichment analysis, and multiple experiments were corrected for FDR. The enriched KEGG pathway is nominally statistically significant at the *p* < 0.05 level.

### Metabolic Assay

The l‐Lactate Assay kit (Jiancheng, China) to measure lactate production, following the manufacturer's protocols was employed. The levels of lactate production were quantified at 450 nm using a microplate reader. ATP production was measured with the ATP assay kit (Jiancheng, China) and glucose consumption was measured with the glucose uptaken assay kit (Abcam, USA) according to the manufacturer's protocol.

### Seahorse Assay

The extracellular flux analyzer, Seahorse XF96 (Agilent Seahorse XF Technology, USA), was used to assess the glycolytic activity of the HCC cells. In Seahorse XF96 well plates, cells were seeded at 12 000 cells per well. Right before the assays, these cells were changed from a culture medium to an assay medium and incubated for 1 h at 37 °C. Under baseline conditions and with the addition of Glucose (Agilent, USA), Oligomycin (Agilent, USA), and 2‐deoxyglucose (Agilent, USA), the extracellular acidification rate (ECAR) was determined. By graphing the total ECAR as a function of time (mpH/min), respectively, in the XF96 Glycolysis report generator, the total extracellular acidification rate (ECAR) was computed. Following the experiment's conclusion, the cells were promptly trypsinized, and the individual well rate data were normalized to protein concentration.

### Co‐Immunoprecipitation (Co‐IP)

To obtain cell lysate, pre‐chilled Lysis buffer (Absin, China) and PMSF (Absin, China) were added to the culture dish. After incubating on ice for 5 min, the cell lysate was transferred to a microcentrifuge tube. Centrifugation at 4 °C, 12000 g for 10 min yielded the supernatant. Subsequently, western blotting was performed to detect the presence of the target protein. Monoclonal antibodies against the target protein (5 µg) were added to 500 µL of cell lysate and incubated overnight at 4 °C. Protein A and Protein G (5 µL each) (Absin, China) were then introduced. Following 1 h of mixing at 4 °C, centrifugation retained the pellet, which was washed three times with Wash buffer (Absin, China). The pellet was resuspended in 40 µL of SDS sample buffer (Absin, China), vortexed, and briefly centrifuged. Finally, Protein A and Protein G were allowed to settle, and the sample was analyzed using western blotting.

### Prediction of Potential Allosteric Sites of GJB2 and Virtual Screening

Using AlphaFold, potential allosteric sites within the GJB2 protein structure were estimated. To identify promising compounds, virtual screening was conducted using two commercial chemical libraries, each containing over 67 000 compounds. The screening process employed grid‐based ligand docking facilitated by the GLIDE software (Schrödinger Maestro 11.4), meticulously targeting the expected locations. Subsequently, the top five compounds with the highest scores from MCE for experimental investigation were procured.

### Animal Model

The Nanjing Medical University Animal Management Committee gave its approval for the animal experiment (IACUC‐2404099), and all experimental techniques and animal care adhered to the institutional ethical standards for animal‐related investigations. The Nanjing Medical University Laboratory Animal Centre used specialised pathogen‐free (SPF) breeding practices to raise all of its mice. The cervical dislocation used to sacrifice mice.

C57BL/6 wild‐type mice were bought from the Nanjing Medical University Model Animal Research Centre. Before tumor implantation, all animals were examined to make sure they were healthy and free of illness. C57BL/6 mice received subcutaneous injections of H22 cells. Four groups were created from the tumor model mice with transplanted carcinomas, sh‐NC+PBS, sh‐GJB2+PBS, sh‐NC+ Anti‐PD1, sh‐GJB2+ Anti‐PD1, each group had five mice. On the eighth day and then twice a week after that, mice were intraperitoneally I.P. injected with 6.6 mg k^−1^g of anti‐PD1 (Bioxcell, USA). In vivo experiments with Sal B, four groups were created from the tumor model mice with transplanted carcinomas, DMSO, Sal B, DMSO+Anti‐PD1, Sal B+ Anti‐PD1, each group had five mice. Specifically, 6.6 mg k^−1^g anti‐PD1 (Bioxcell, USA) were intraperitoneally I.P. injected into mice on the eighth day and then twice a week. 10 mg k^−1^g Sal B (MCE, USA) were intraperitoneally I.P. injected into mice on the seventh day, and every other day for two weeks.

### Mass Cytometry

The detailed experimental methods are shown in our previous article.^[^ ^33^ ^]^

### Microscale Thermophoresis (MST)

With the use of conventional capillaries and Monolith NT.115 (NanoTemper Technologies GmbH, Germany), MST was used to ascertain the binding affinity of GJB2 with Salvianolic acid B. Lysates from HEK293T cells expressing GFP‐GJB2 were used as a source of fluorescently labeled GJB2. HEK293T cells were transfected with GFP‐fused GJB2 and lysed 24 h after transfection. To assess the binding of Salvianolic acid B to GJB2, cell lysates were diluted 1.5‐fold with MST buffer (10 mm Na‐phosphate buffer, pH 7.4, 1 mm MgCl2, 3 mm KCl, 150 mm NaCl, 0.05% Tween‐20) to provide optimal fluorescence levels. Salvianolic acid B titration series (0–1 mm) was incubated with diluted cell lysates. Measurements were performed in a high‐quality coated capillary (NanoTemper Technologies GmbH, Germany) using an LED light source at 470 nm with 100% IR laser power and a temperature of 25 °C. The fluorescence signal was normalized, and the Hill equation was fitted using MO Affinity Analysis v2.1.3 software (NanoTemper Technologies GmbH, Germany).

### Statistical Analysis

Most of the analyses in this study were performed using Graphpad Prism 10.0 with *p*‐value of 0.05 for statistical significance. The *t*‐test was used to analyze the differences between the two sample groups. One‐way ANOVA was used to analyze the differences between three or more sample groups. Detailed data on the results of all analyses are available in Figure legends.

### Ethics Approval and Consent To Participate

This study, which involves human participants, received approval from the Ethics Committee of Nanjing Medical University and Zhengzhou University (2019‐SRFA‐238, 2020‐SRFA‐314). All in vivo animal experiments were approved by the Committee on the Ethics of Animal Experiments of Nanjing Medical University (IACUC‐2404099).

## Conflict of Interest

The authors declare no conflict of interest.

## Author Contributions

H.Y.L., X.L., C.W.Z., and X.P.H. contributed equally to this work and are co‐first authors. Dr. H.Y.L., X.L., C.W.Z., and X.P.H. were responsible for designing and performing the experiments. Dr. Y.F.C., H.Z., Y.L.W., N.Y., T.H., C.L., H.S.C., Z.Q.L. contributed to performing part of experiment. Furthermore, there are five corresponding authors in this manuscript. Dr. J.H.S., L.L., H.J.W., Z.X.L. and W.W.T. have contributed to study design and critical revision of the manuscript. All authors read and approved the final manuscript.

## Supporting information

Supporting Information

## Data Availability

The data that support the findings of this study are available from the corresponding author upon reasonable request.
