# Supplementary material for: GJB2 Promotes HCC Progression by Activating Glycolysis Through Cytoplasmic Translocation and Generating a Suppressive Tumor Microenvironment Based on Single Cell RNA Sequencing
Source: Adv Sci (Weinh). 2024 Aug 20;11(39):2402115. doi: 10.1002/advs.202402115 (PMC11497106; doi:10.1002/advs.202402115)
Supplement: Supplementary file 1 — Supporting Information [file ADVS-11-2402115-s001.docx]

**GJB2 promotes HCC progression by activating glycolysis through cytoplasmic translocation and generating a suppressive tumor microenvironment based on single cell RNA sequencing**

**Supplementary figures**


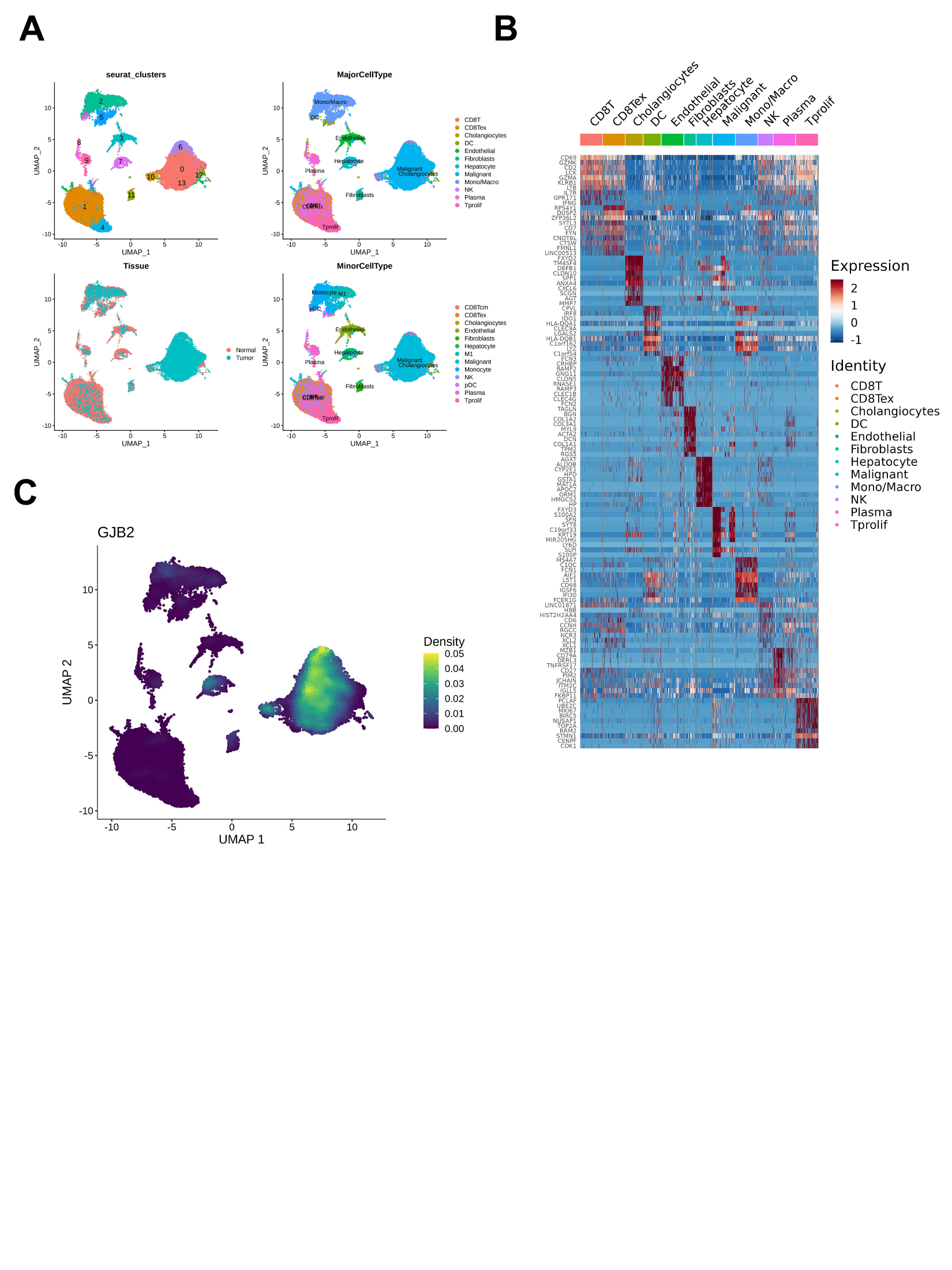


**Figure S1**

(A) UMAP plots show the different cell distributions in the GSE138709 database (intrahepatic cholangiocarcinoma).

(B) Heat map shows the specific genes expressed by the cell clusters in Figure S1A.

(C) UMAP plot showed the relative expression of GJB2 in different cell clusters, and the results showed that GJB2 was highly expressed in malignant cells.


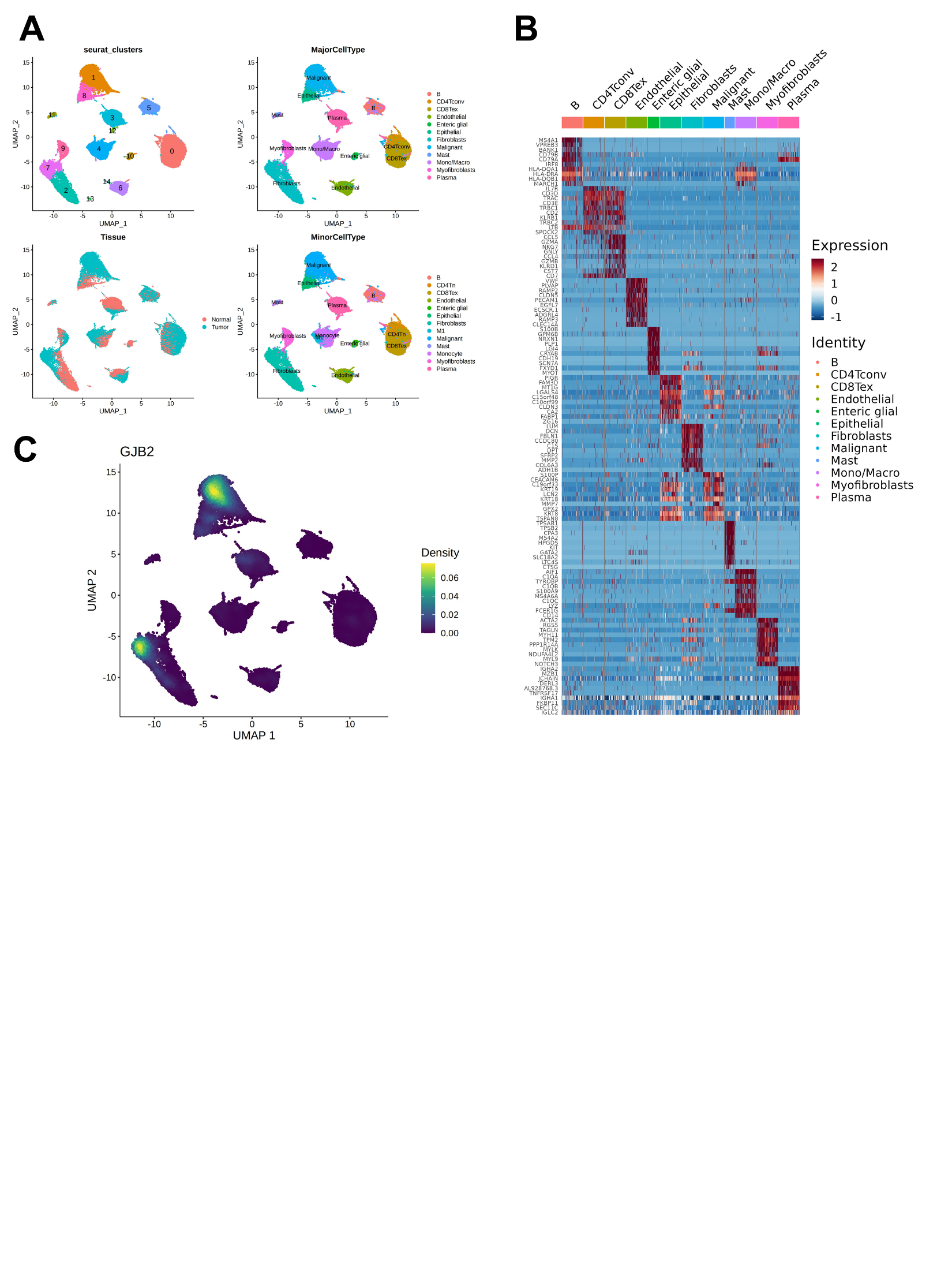


**Figure S2**

(A) UMAP plots show the different cell distributions in the EMTAB8107 database (colorectal carcinoma).

(B) Heat map shows the specific genes expressed by the cell clusters in Figure S2A.

(C) UMAP plot showed the relative expression of GJB2 in different cell clusters, and the results showed that GJB2 was highly expressed in malignant cells.


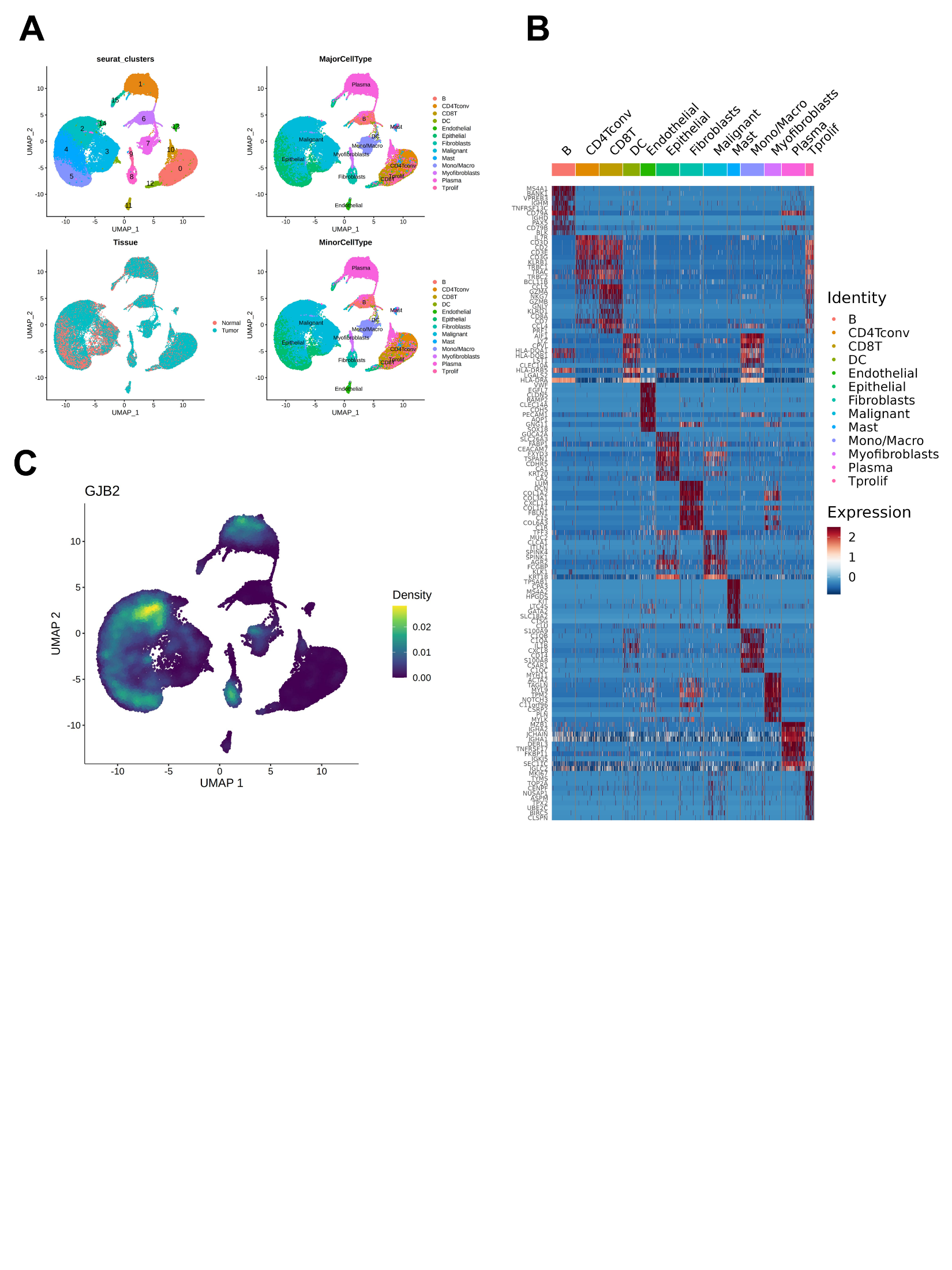


**Figure S3**

(A) UMAP plots show the different cell distributions in the GSE166555 database (colorectal carcinoma).

(B) Heat map shows the specific genes expressed by the cell clusters in Figure S3A.

(C) UMAP plot showed the relative expression of GJB2 in different cell clusters, and the results showed that GJB2 was highly expressed in malignant cells.


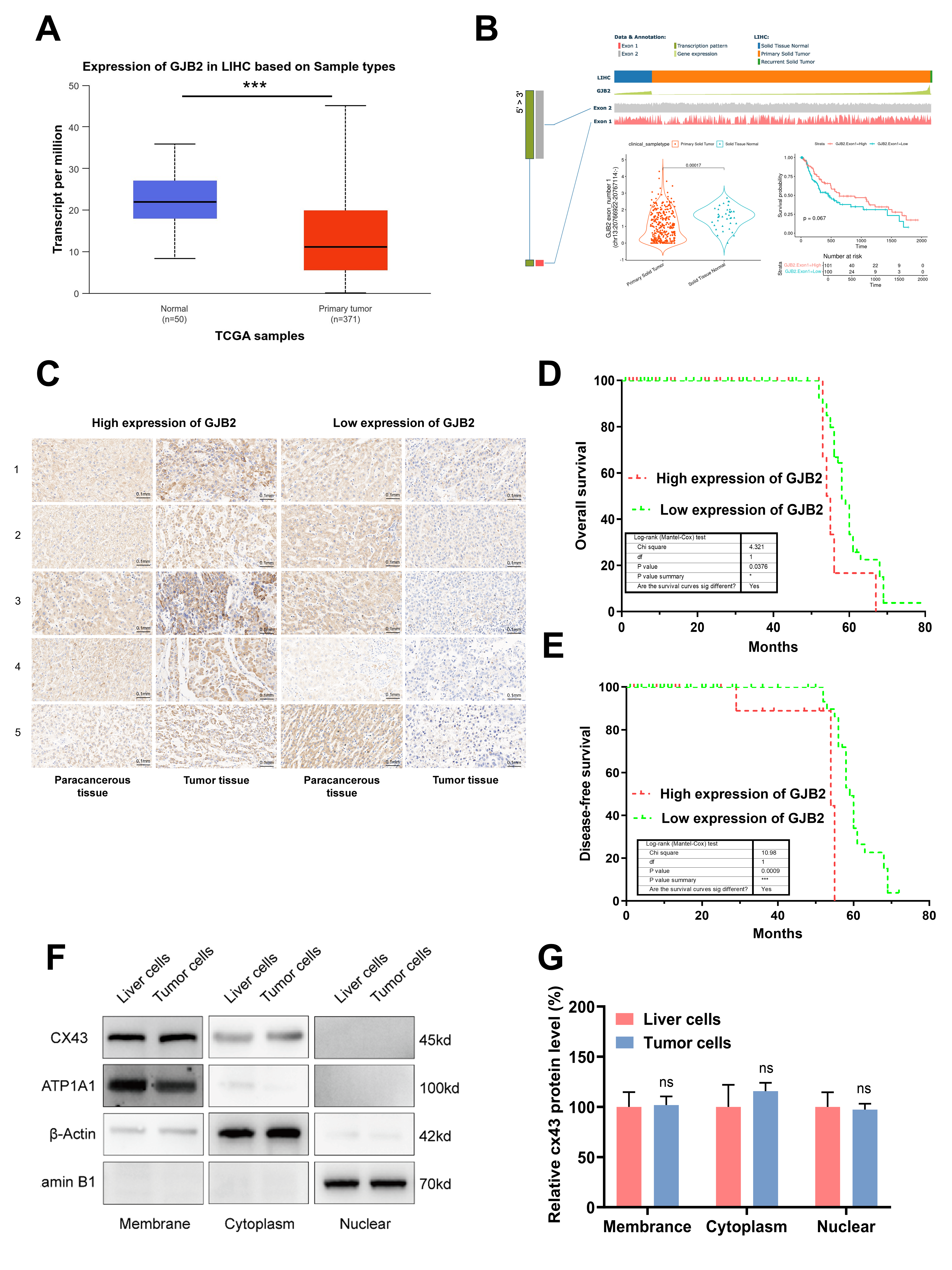


**Figure S4**

(A) The TCGA database showed the expression of GJB2 mRNA in HCC cancer tissues (n=371) and adjacent tissues (n=50).

(B) The TSVDB database shows that GJB2 has two exons, exon1(short) near the 5 'end and exon2(long) near the 3' end. The violin diagram showed that exon1 expression in HCC tissue (n=371) was lower than that in normal liver tissue (n=50). (p=0.00017). Survival analysis of patients with low exon1 expression (n=136) and high exon1 expression (n=172) in HCC showed that patients with low exon1 expression in GJB2 in HCC had worse prognosis. (p=0.067).

(C) GJB2 expression was detected in 109 human HCC tissues and adjacent normal tissues by immunohistochemistry, and 5 representative samples with high GJB2 expression and low GJB2 expression were selected. (Scale bar, 0.1mm).

(D-E) Kaplan-Meier survival curves of relapse-free survival (p=0.0009) and overall survival (p=0.0376) in HCC patients with high GJB2 expression and those with low GJB2 expression were statistically analyzed using Log-rank tset.

(F-G) Expression of cx43 protein in cell membrane, cytoplasm and nucleus of HCC cells and liver cells in mouse organoids. β-actin is used for loading control of cytoplasmic proteins, ATP1A1 is used for loading control of cytoplasmic proteins and Lamin B1 is used for loading control of nuclear protein. n=3 independent biological replicates.

In all statistical plots, data are expressed as the mean ± SD, Student’s t test (Figure S4A, F) and Log-rank tset (Figure S4C, D) were used to determine statistical significance. (ns=not significant, *P < 0.05, **P < 0.01, ***P < 0.001).


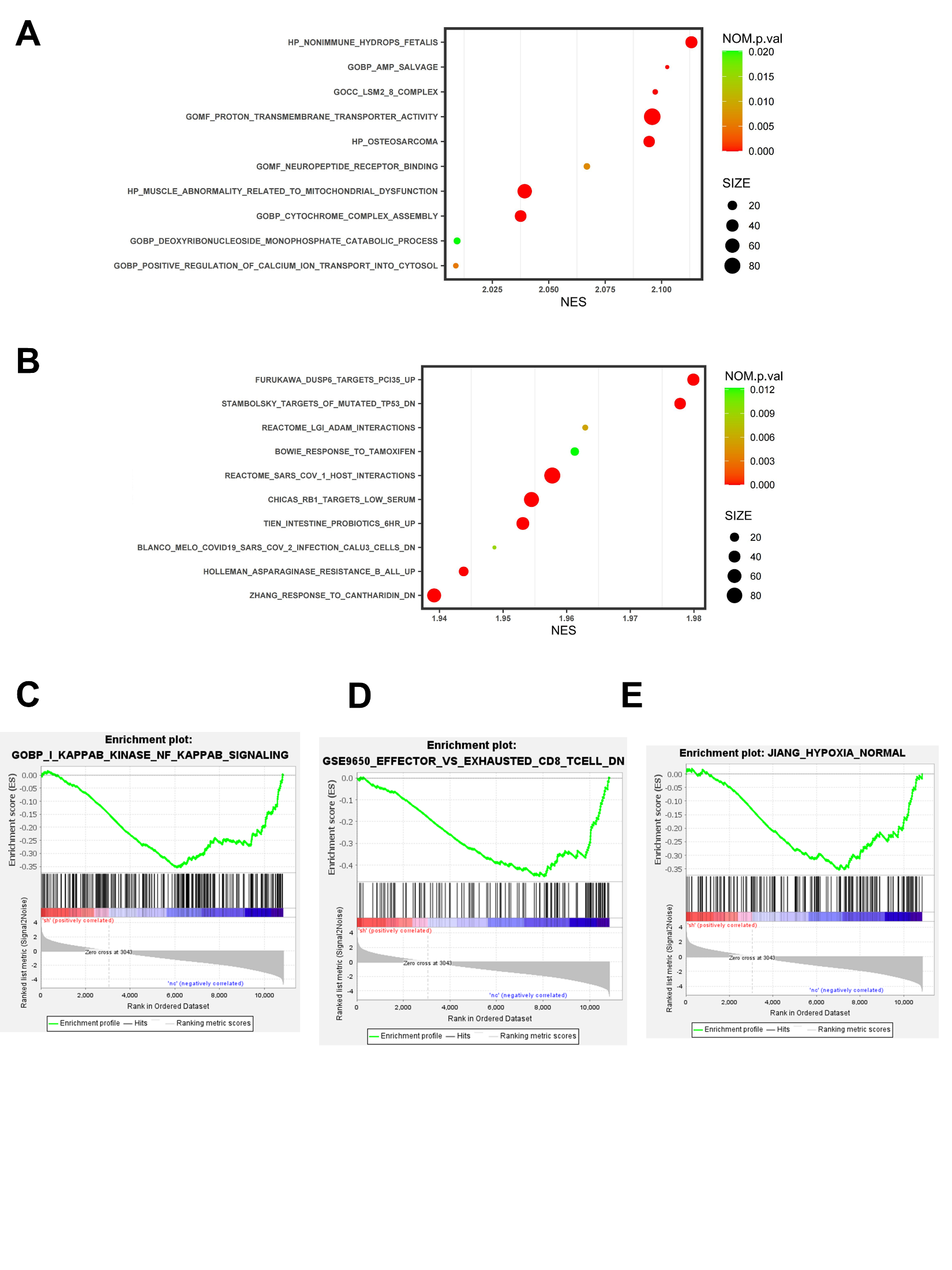


**Figure S5**

(A-B) As revealed by KEGG analysis results, TP53 mutations were among the most obviously involved processes.

(C-E) Gene set enrichment analysis (GSEA) suggested that the down-regulated genes are associated with NF-κB, CD8+ T exhaustion, and hypoxia pathway.


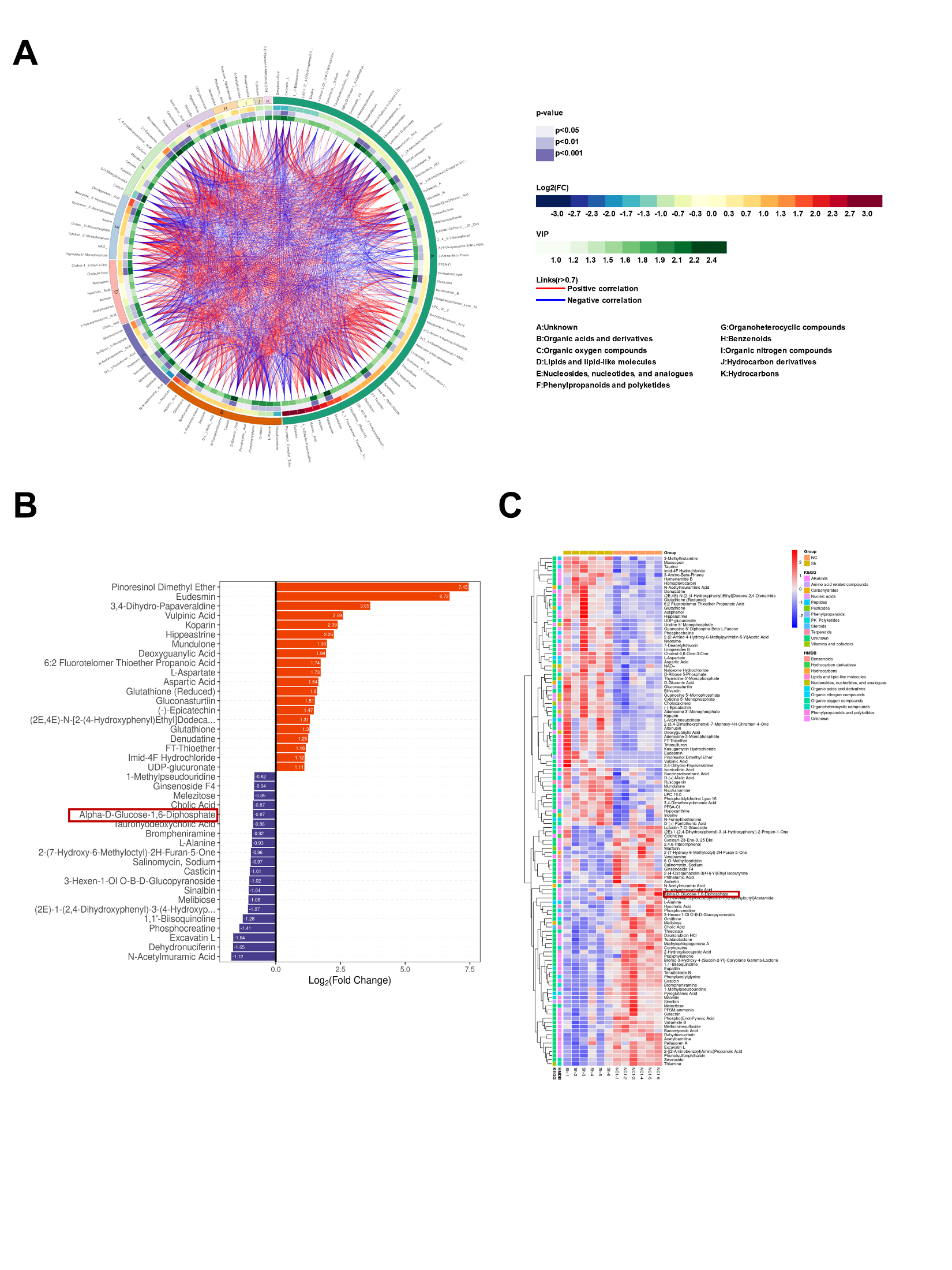


**Figure S6**

(A) The results of metabolomics were analyzed by mixed-mode analysis. The cycle diagram mainly showed the correlation between multiple differential metabolites.

(B) The results of metabolomics were analyzed by mixed-mode analysis. The top up-regulated metabolites after GJB2 were knockdown in HCC cells.

(C) The results of metabolomics were analyzed by mixed-mode analysis. The different metabolites in each comparison group were classified and counted according to the structure and function of the metabolites, and the results of substance classification in the KEGG and HMDB databases were provided respectively.


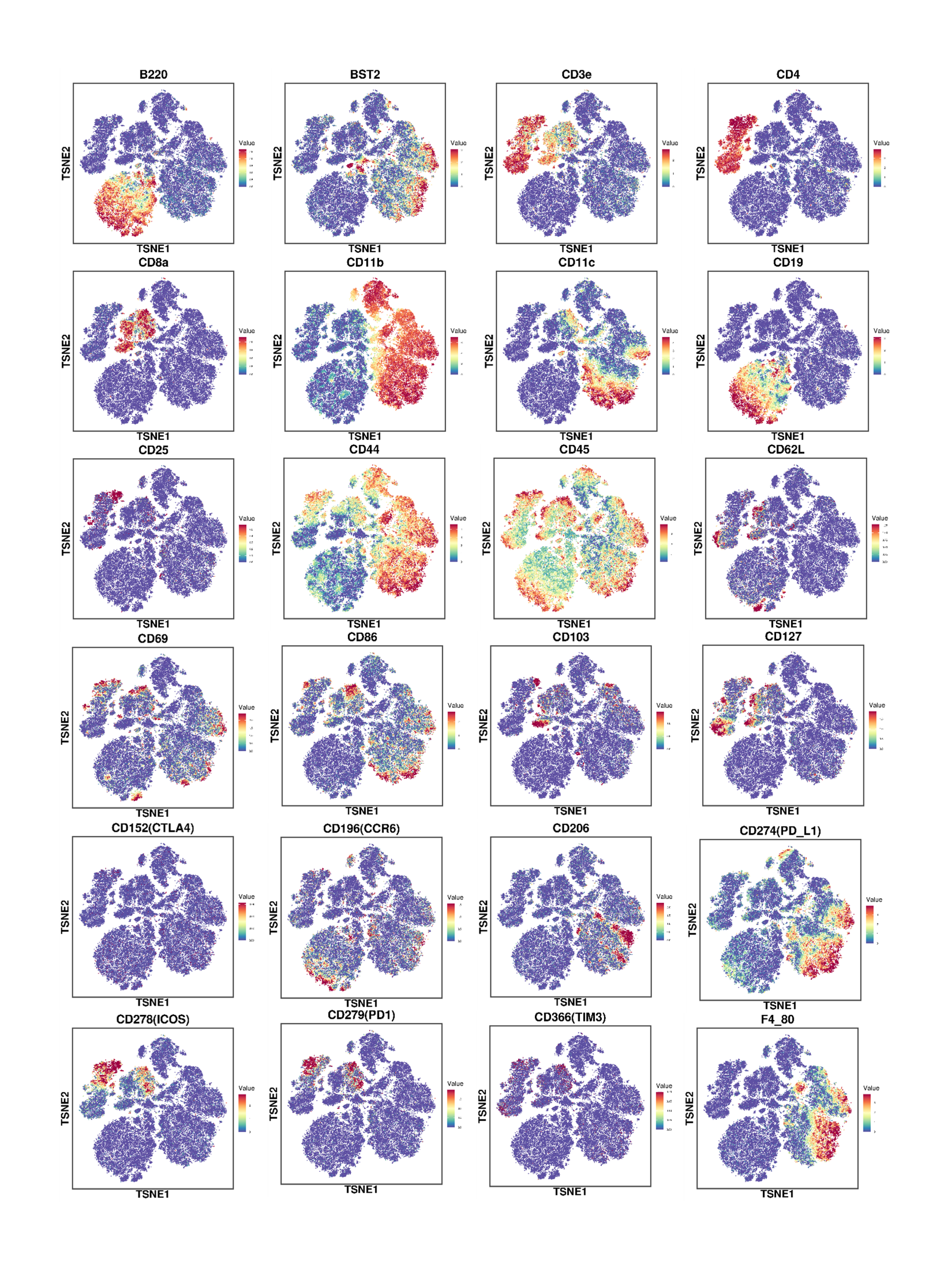


**Figure S7**

the respective cell clusters were defined based on specific markers for the respective cell type.


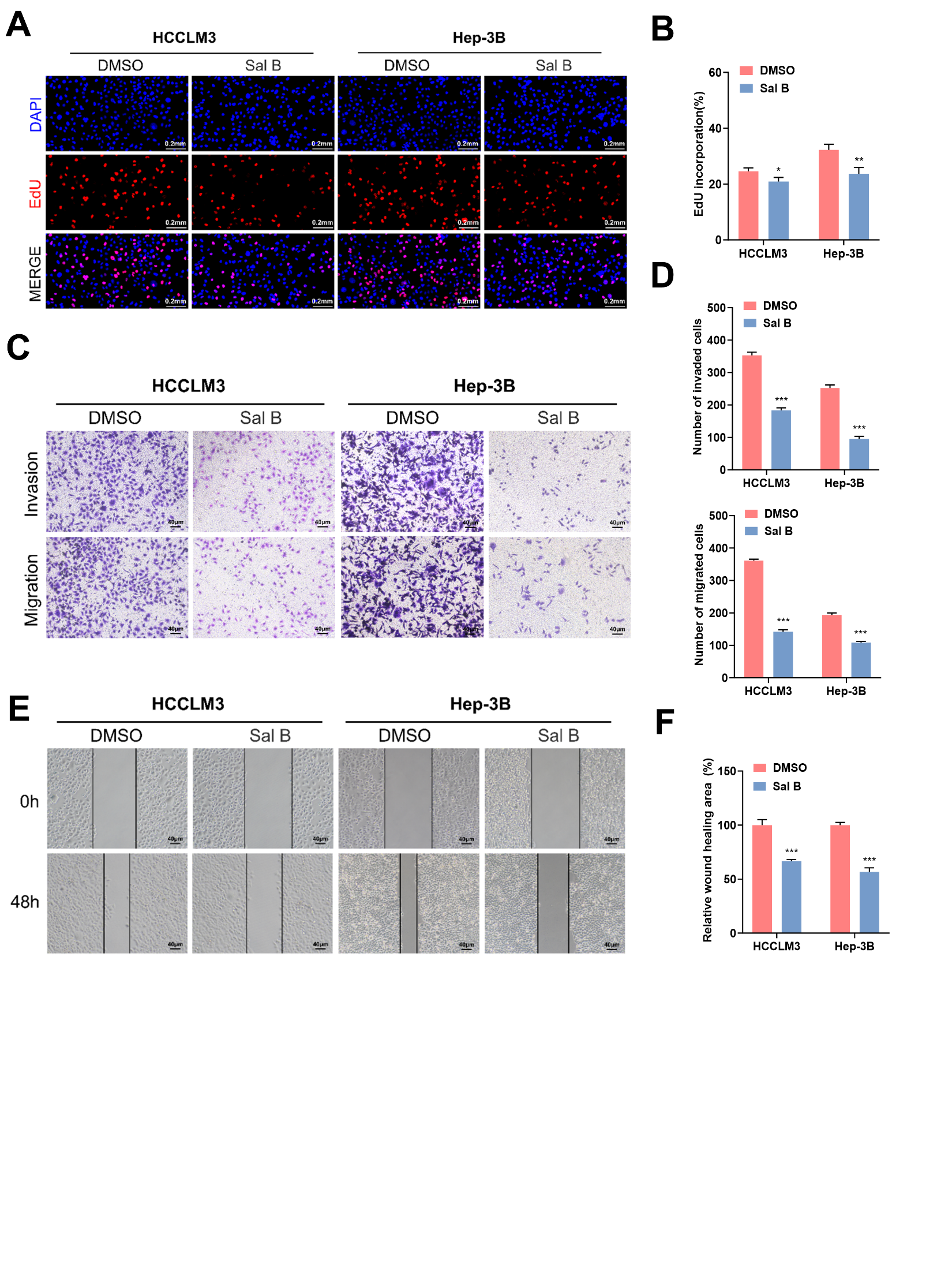


**Figure S8**

(A-B) EdU assays were performed to assess the effect of Salvianolic acid B on HCC cell proliferation (scale bars, 0.2mm). n=3 independent biological replicates.

(C-D) Transwell assays were performed to determine the migration and invasion capacities of HCC cell lines (scale bars, 40 μm). n=3 independent biological replicates.

(E-F) The effect of Salvianolic acid B on HCC cell migration was evaluated through wound healing assays (scale bars, 40μm). n=3 independent biological replicates.

In all statistical plots, data are expressed as the mean ± SD, Student’s t test (Figure S4B, D, F) were used to determine statistical significance. (ns=not significant, *P < 0.05, **P < 0.01, ***P < 0.001).

**Supplementary tables**

**Table S1. Clinical characteristics of 109 patients with HCC**

| **Characteristic** | | **Total**  **(cases [%])** | **GJB2 relative expression (cases [%])** | | ***P* value** |
| --- | --- | --- | --- | --- | --- |
|  |  |  | **Low (*n* = 62)** | **High (*n* = 47)** |  |
| **Age** | <60 years | 70 (64.2) | 41 (58.6) | 29 (41.4) | 0.391 |
|  | ≥60 years | 39 (35.8) | 21 (53.8) | 18 (46.2) |  |
| **Gender** | Male | 91 (83.4) | 53 (58.2) | 38 (41.8) | 0.348 |
|  | Female | 18 (16.6) | 9 (50.0) | 9 (50.0) |  |
| **Tumor size** | <5 cm | 41 (37.6) | 26 (63.4) | 15 (36.6) | 0.192 |
|  | ≥5 cm | 68 (62.4) | 36 (53.0) | 32 (47.0) |  |
| **Microvascular invasion** | Absent | 62 (56.9) | 42 (67.8) | 20 (32.2) | 0.007 * |
|  | Present | 47 (43.1) | 20 (42.6) | 27 (57.4) |  |
| **Blood vessel invasion** | Absent | 75 (68.8) | 48 (64.0) | 27 (36.0) | 0.022 * |
|  | Present | 34 (31.2) | 14 (41.2) | 20 (58.8) |  |
| **Macrovascular invasion** | Absent | 89 (81.7) | 57 (64.1) | 32 (35.9) | 0.002 * |
|  | Present | 20 (18.3) | 5 (25.0) | 15 (75.0) |  |
| **TNM stage** | I-II | 68 (62.4) | 42 (61.8) | 26 (38.2) | 0.130 |
|  | III-IV | 41 (37.6) | 20 (48.8) | 21 (51.2) |  |

*P < 0.05

| **Table S2. Primer sequences, shRNAs used in this study** | | |
| --- | --- | --- |
| **Primer/shRNA** | | **Sequence (5’-3’)** |
| human-GJB2 | Forward primer | GTCCGTGACAGCGTTTTTCTT |
|  | Reverse primer | AATTGAGGCTGCTACGTTTATGT |
| human-GLUT1 | Forward primer | ATCGGTTCAAGTATGCTCGGG |
|  | Reverse primer | GACCAAGTTTTCCACGACGTT |
| human-LDHA | Forward primer | ATGTCTGGCTTGCACCTAGTA |
|  | Reverse primer | CCCCAAAGCGAGTAACAAATTCT |
| human-HIF-1α | Forward primer | GCCCCTCTACTTGGAAGACGA |
|  | Reverse primer | AAGTGATCCCATACAGGGCTC |
| human-HK2 | Forward primer | GAGCCACCACTCACCCTACT |
|  | Reverse primer | CCAGGCATTCGGCAATGTG |
| human-PKM2 | Forward primer | ATGTCGAAGCCCCATAGTGAA |
|  | Reverse primer | TGGGTGGTGAATCAATGTCCA |
| human-PDK1 | Forward primer | GAGAGCCACTATGGAACACCA |
|  | Reverse primer | GGAGGTCTCAACACGAGGT |
| human-PD-L1 | Forward primer | GCCCCTCTACTTGGAAGACGA |
|  | Reverse primer | AAGTGATCCCATACAGGGCTC |
| human-IκBα | Forward primer | AAGTGATCCGCCAGGTGAAG |
|  | Reverse primer | CTGCTCACAGGCAAGGTGTA |
| human-sh1-GJB2 | Forward | ccggGCATTATGATCCTCGTTGTGGctcgagCCACAACGAGGATCATAATgctttttg |
|  | Reverse | aattcaaaaaGCATTATGATCCTCGTTGTGGctcgagCCACAACGAGGATCATAATGC |
| human-sh2-GJB2 | Forward | ccggGCATCTTCTTCCGGGTCATCTctcgagAGATGACCCGGAAGAAGATgctttttg |
|  | Reverse | aattcaaaaaGCATCTTCTTCCGGGTCATCTctcgagAGATGACCCGGAAGAAGATGC |
| human-sh3-GJB2 | Forward | ccggGCATCCTGCTGAATGTCACTGctcgagCAGTGACATTCAGCAGGATgctttttg |
|  | Reverse | aattcaaaaaGCATCCTGCTGAATGTCACTGctcgagCAGTGACATTCAGCAGGATGC |
| mouse-sh1-GJB2 | Forward | gatccGCTATTTGTTCGTTAGGTATTtcaagagAATACCTAACGAACAAATAGCtttttt |
|  | Reverse | aattaaaaaaGCTATTTGTTCGTTAGGTATTctcttgaAATACCTAACGAACAAATAGCg |
| mouse-sh2-GJB2 | Forward | gatccGTCTGGTGAAATGCAACGCTTtcaagagAAGCGTTGCATTTCACCAGACtttttt |
|  | Reverse | aattaaaaaaGTCTGGTGAAATGCAACGCTTctcttgaAAGCGTTGCATTTCACCAGACg |
| mouse-sh3-GJB2 | Forward | gatccGAGAGATAAAGAACGAGTTTAtcaagagTAAACTCGTTCTTTATCTCTCtttttt |
|  | Reverse | aattaaaaaaGAGAGATAAAGAACGAGTTTActcttgaTAAACTCGTTCTTTATCTCTCg |
| His-GJB2-WT | CTCGAGGCCACCatggattggggcacgctgcagacgatcctggggggtgtgaacaaacactccaccagcattggaaagatctggctcaccgtcctcttcatttttcgcattatgatcctcgttgtggctgcaaaggaggtgtggggagatgagcaggccgactttgtctgcaacaccctgcagccaggctgcaagaacgtgtgctacgatcactacttccccatctcccacatccggctatgggccctgcagctgatcttcgtgtccacgccagcgctcctagtggccatgcacgtggcctaccggagacatgagaagaagaggaagttcatcaagggggagataaagagtgaatttaaggacatcgaggagatcaaaacccagaaggtccgcatcgaaggctccctgtggtggacctacacaagcagcatcttcttccgggtcatcttcgaagccgccttcatgtacgtcttctatgtcatgtacgacggcttctccatgcagcggctggtgaagtgcaacgcctggccttgtcccaacactgtggactgctttgtgtcccggcccacggagaagactgtcttcacagtgttcatgattgcagtgtctggaatttgcatcctgctgaatgtcactgaattgtgttatttgctaattagatattgttctgggaagtcaaaaaagccagttCATCATCACCATCACCATGGGAATTC | |
| His-GJB2-MUT | CTCGAGGCCACCatggattggggcacgctgcagacgatcctggggggtgtgaacaaacactccaccagcattaaggaggtgtggggagatgagcaggccgactttgtctgcaacaccctgcagccaggctgcaagaacgtgtgctacgatcactacttccccatctcccacatccggagacatgagaagaagaggaagttcatcaagggggagataaagagtgaatttaaggacatcgaggagatcaaaacccagaaggtccgcatcgaaggctcctatgtcatgtacgacggcttctccatgcagcggctggtgaagtgcaacgcctggccttgtcccaacactgtggactgctttgtgtcccggcccacggagaagactgtcttcacaagatattgttctgggaagtcaaaaaagccagttCATCATCACCATCACCATGGGAATTC | |

| **Table S3. The associated antibodies used in this study** | |
| --- | --- |
| **Name** | **Catalog Number** |
| GJB2 | 16960-1-AP, proteintech, China |
| IKKα/β | ab178870, abcam, USA |
| p-IKKα/β (Ser176/180) | #2697, Cell Signaling Technology, USA |
| IκBα | 10268-1-AP, proteintech, China |
| p-IκBα (Ser32/36) | #9246, Cell Signaling Technology, USA |
| P65 | 10745-1-AP, proteintech, China |
| p-P65 (Ser536) | #3033, Cell Signaling Technology, China |
| ubiquitin | 10201-2-AP, proteintech, China |
| HIF-1α | 20960-1-AP, proteintech, China |
| GLUT-1 | 21829-1-AP, proteintech, China |
| ASB2 | ab200370, abcam, USA |
| PD-L1 | 66248-1-Ig, proteintech, China |
| CD8 | 66868-1-Ig, proteintech, China |
| CD86 | 13395-1-AP, proteintech, China |
| CD163 | 16646-1-AP, proteintech, China |
| KI67 | 27309-1-AP, proteintech, China |
| β-Actin | 66009-1-Ig, proteintech, China |
| Affinipure Goat Anti-Rabbit IgG | SA00001-2, proteintech, China |
| Affinipure Goat Anti-Mouse IgG | SA00001-1, proteintech, China |
| His-Tag | 66005-1-Ig, proteintech, China |
| ATP1A1 | 14418-1-AP, proteintech, China |
| Lamin B1 | 12987-1-AP, proteintech, China |
| Connexin 43 | 26980-1-AP, proteintech, China |
| CD86 | 13395-1-AP, proteintech, China |
| CD163 | 16646-1-AP, proteintech, China |
| Goat Anti-Mouse IgG Alexa Fluor 555 | A0460, Beyotime, China |
| Donkey Anti-Rabbit IgG Alexa Fluor 555 | A0453, Beyotime, China |
